# Design and Synthesis of Matrine Derivatives as Novel Anti-Pulmonary Fibrotic Agents via Repression of the TGFβ/Smad Pathway

**DOI:** 10.3390/molecules24061108

**Published:** 2019-03-20

**Authors:** Lingyu Li, Liyan Ma, Dongchun Wang, Hongmei Jia, Meng Yu, Yucheng Gu, Hai Shang, Zhongmei Zou

**Affiliations:** 1 Institute of Medicinal Plant Development, Chinese Academy of Medical Sciences and Peking Union Medical College, Beijing 100193, China; bluehill07@163.com (L.L.); lyma@implad.ac.cn (L.M.); m18811455180@163.com (D.W.); hmjia@implad.ac.cn (H.J.); myu@implad.ac.cn (M.Y.); 2 Syngenta, Jealott’s Hill International Research Centre, Berkshire, Bracknell RG42 6EY, UK; yucheng.gu@syngenta.com; 3 State Key Laboratory of Bioactive Substance and Function of Natural Medicines, Institute of Materia Medical, Chinese Academy of Medical Sciences and Peking Union Medical College, Beijing 100050, China

**Keywords:** idiopathic pulmonary fibrosis, matrine, indole, structure activity relationship, fibroblast-to-myofibroblast transition, TGF-β/Smad pathway

## Abstract

A total of 18 matrine derivatives were designed, synthesized, and evaluated for their inhibitory effect against TGF-β1-induced total collagen accumulation in human fetal lung fibroblast MRC-5 cell lines. Among them, compound **3f** displayed the most potent anti-fibrotic activity (IC_50_ = 3.3 ± 0.3 μM) which was 266-fold more potent than matrine. **3f** significantly inhibited the fibroblast-to-myofibroblast transition and extracellular matrix production of MRC-5 cells. The TGF-β/small mothers against decapentaplegic homologs (Smad) signaling was also inhibited by **3f**, as evidenced by inhibition of cytoplasm-to-nuclear translocation of Smad2/3 and suppression of TGF-β1-induced upregulation of TGF-β receptor type I (TGFβRI). Additionally, **3f** exhibited potent inhibitory effects against TGF-β1-induced fibroblasts migration. These data suggested that **3f** might be a potential agent for the treatment of idiopathic pulmonary fibrosis via repression of the TGFβ/Smad signaling pathway.

## 1. Introduction

Idiopathic pulmonary fibrosis (IPF) is a progressive and irreversible lung disease characterized by abnormally excessive accumulation of extracellular matrix (ECM) [1]. It is associated with high morbidity and mortality, with a median survival ranging from only three to five years from diagnosis [2]. Unfortunately, treatments for IPF were quite limited, and to date, only two small molecule drugs (Pirfenidone and Nintedanib) were approved by FDA for IPF treatment; nonetheless their treatment effect was not satisfactory [3,4]. Therefore, novel anti-IPF agents were urgently needed.

Matrine, a pyrrolizidine alkaloid isolated from traditional Chinese medicine *Sophora flavescens*, attracted our interest for its proved anti-fibrotic activities. Yu et al. [5] demonstrated that matrine exhibited significant inhibitory effect against liver fibrosis by reducing the expression of transforming growth factor-β1 (TGF-β1) and enhancing the activity of hepatocyte growth factor. The anti-fibrotic activities of matrine on cardiac fibrosis via inhibition of TGF-β/Smad pathway [6] or affection of ATF6 signaling pathway [7] had also been suggested by previous studies. In addition, two independent clinical studies [8,9] revealed that 150 mg/d matrine in combination of prednisone could significantly improve lung function and reduce symptom integral of the patients. Clinical study [10] also demonstrated that oxymatrine, the *N*-oxide analogue of matrine, could significantly reduce serum matrix metalloproteinase-9 (MMP-9) and TGF-β1 level in IPF patients. Collectively, matrine is of great potency for IPF treatment and worthy of further investigation.

**Figure 1:**
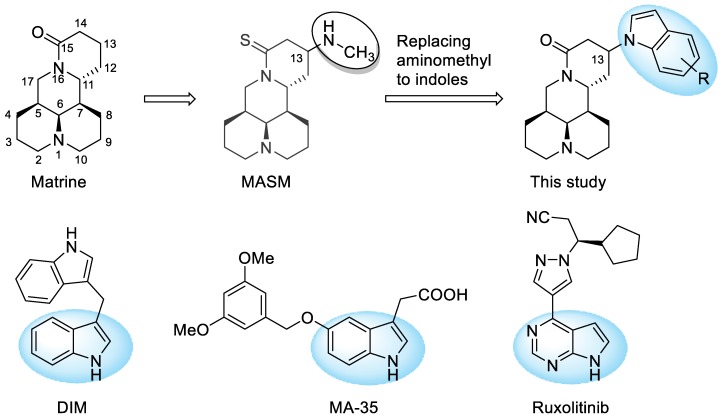
Chemical structures of matrine, lead compound MASM, indole-containing anti-fibrotic drug or candidates, and the structure modification strategy.

MASM (Figure 1) was a matrine derivative discovered by Xu et al. [11] which exhibited potent anti-fibrotic activities. Their research also suggested that the introduction of alkyl amine groups at the 13-position of matrine had a significant impact on anti-liver fibrosis [11] and anti-inflammatory activities [12]. Taking MASM as a lead, we changed the thioamide motif back to the original amide structure of matrine due to the water-instability of MASM [13], and performed structural optimization by varying the alkylamino substituent groups at the 13-position of matrine.

Indole is a privileged scaffold in anti-fibrotic drug discovery. For instance, Xia and coworkers [14] demonstrated that indole derivative DIM could attenuate renal fibrosis via inhibiting fibroblast-to-myofibroblast transition and Smad2/3 phosphorylation. Additionally, in an anti-fibrotic screening of indole compound library, Shima et al. [15] observed that MA-35 displayed potent anti-fibrotic activities via inhibition of TGF-β1 signaling and suppression of ECM production. Moreover, indole scaffold could also be found in the chemical structure of anti-fibrotic drug Ruxolitinib [16]. Therefore, a series of 13-indolyl substituted derivatives was also designed and synthesized. Herein, we describe the synthesis of 18 matrine derivatives, SAR analysis, as well as the primary mechanism of action of the key compound.

## 2. Results and Discussion

### 2.1. Chemistry

**Scheme 1:**
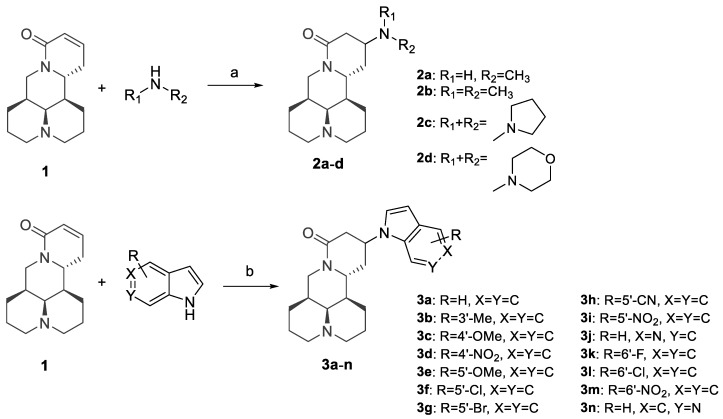
Reagents and conditions: (**a**) H_2_O, 100 W microwave irradiation, 30 min; (**b**) KHMDS, THF, −20 °C to rt, overnight.

The synthetic routes of all target compounds were outlined in Scheme 1. The aliphatic amine substituted derivatives **2a**–**d** were synthesized by microwave-assisted water-mediated Aza-Michael addition. Reactions of aliphatic amines and sophocarpine (**1**), a natural dehydrogenation analogue of matrine, proceeded smoothly under microwave irradiation to give desired products in yields of 90%–95%. Then, a series of indole substituted derivatives **3a**–**n** were obtained by Michael addition of sophocarpine and indole in the presence of KHMDS in yields of 35–77%. All of the desired products were purified with silica gel chromatography using dichloromethane/methanol as eluents.

**Figure 2:**
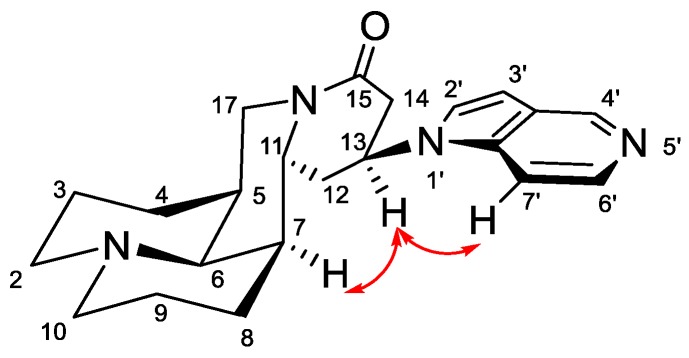
Key NOE correlations of **3j**.

The Michael addition reaction introduced a new chiral center at the 13 position of matrine. Previous literature [12] determined the configuration of 13-position to be *13S*, however, no experimental evidence supporting this determination was reported. In this study, we found that most of the synthesized compounds appeared in pairs of 13*S*/13*R* stereoisomers, of which the 13*S*-isomerswere the major stereoisomers. Nonetheless, four compounds (**2a**, **2b**, **3f**, **3j**) appeared in pure 13*S* form, and we determined the configuration of 13 position based on NOE experiment of **3j** (See Supplementary Materials). As illustrated in Figure 2, we observed NOE correlation between H-13 and H-7, indicating that these protons were in the same face. Thus, we confirmed that the major stereoisomers were 13*S*-isomers.

### 2.2. Inhibition of TGF-β1-Induced Collagen Accumulation by Target Compounds

Excessive accumulation of ECM in the alveolar parenchyma and progressive scarring of lung tissue are major characteristics of IPF [17]. TGF-β1 is the most potent inducer of ECM production as well as the most significant fibrotic cytokines characterized so far [18]. It promotes IPF disease progression by inducing differentiation of fibroblast to myofibroblast and upregulating the secretion of ECM proteins [18,19], especially collagen, which is the most abundant protein in ECM [20]. Therefore, we established an in vitro anti-fibrotic screening model based on TGF-β1-induced collagen accumulation in human fetal lung fibroblasts (MRC-5 cell lines). The inhibitory effect against collagen accumulation of the synthesized compounds were quantitively determined by Sircol collagen assay, a colorimetric bioassay which was also adopted by Intermune incorporation (the innovator of Pirfenidone) for anti-fibrotic screening of Pirfenidone and its derivatives [21]. Cytotoxicity of all compounds were also determined against MRC-5 cells, so as to calculate selectivity index (SI, CC_50_/IC_50_). Higher SI values indicates better safety profile. The structures and activity data were summarized in Table 1. Pirfenidone was used as positive control, and its anti-fibrotic IC_50_ value determined in this study was 1320 ± 98 μM, which was consistent with Intermune’s results (23–69% inhibition of collagen accumulation at 1000 μM concentration for Pirfenidone derivatives [21]).

The SAR investigation was initiated with variation of the alkylamino substituent groups at the 13 position of matrine, by which four derivatives were obtained (**2a**–**d**). As shown in Table 1, the IC_50_ values of **2a**–**d** were 65.3 μM to 255.8 μM, which improved the potency of matrine by 3- to 13-folds, corroborating that 13 position substitution was beneficial for anti-pulmonary fibrosis activity. However, it seemed that the anti-fibrotic potency was largely influenced by steric effect of alkylamino substituents, and smaller substituent was favored. Introduction of bulky substituents such as cycloalkylamino group (**2c**–**d**) or dimethylamino group (**2b**) resulted in 1.4- to 3.9-fold decrease in anti-fibrotic potency compared to methylamino substituted derivative (**2a**). As a consequence, the steric influence in alkylamino substituted derivatives limited the scope for further improvement.

Next, we sought to replace the alkylamino substituents of the 13 position to indolyl group, and 14 derivatives were obtained (**3a**–**n**). To our delight, most of the compounds displayed more significant improvement in anti-fibrotic potency than matrine. Among them, compounds **3d**, **3f**, and **3g** gave inspiring IC_50_ values of 4.3 μM, 3.3 μM, and 6.5 μM, respectively, which was 204-fold, 266-fold, and 135-fold more potent than matrine; or 307-fold, 400-fold, and 203-fold more potent than Pirfenidone. Compounds **3d** and **3f** also showed marked improvement in SI over matrine (from 2.8 to 7.6–8.0). 

Further analysis of **3a**–**n** suggested a remarkable impact of electronic properties of indolyl substitution on anti-fibrotic potency. Compounds with strong electron withdrawing groups such as halogen (**3f**–**g**, **3k**–**l**) or nitro group (**3d**, **3i**, **3m**) gave more potent IC_50_ values (3.3–27.0 μM), an exception being compound **3h** with cyano group whose IC_50_ was 72.1 μM. Contrarily, the introduction of electron donating groups resulted in significant loss of anti-fibrotic potency, and the IC_50_ values of methyl (**3b**) or methoxy (**3c**, **3e**) substituted compounds were around 39.0 to 68.1 μM. 

**Figure 3:**
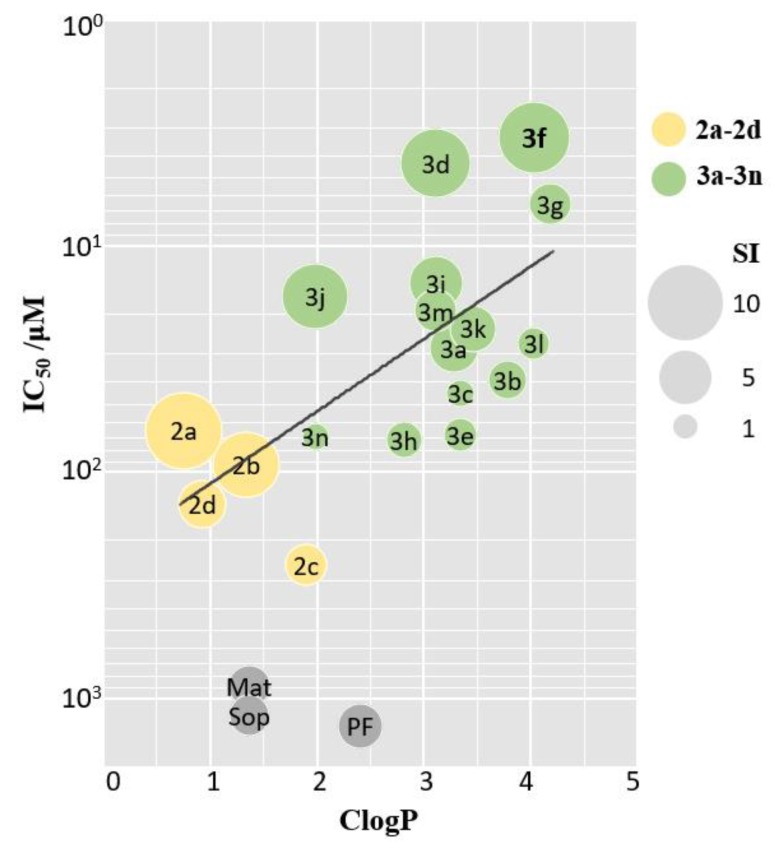
Bubble chart of ClogP-IC_50_. X-axis and Y-axis represents ClogP and IC_50_ values, respectively. Bubble size reflects SI value. Yellow, green, and grey bubbles represent derivatives **2a**–**d**, **3a**–**n**, and positive control, respectively. Mat, Sop, PF was abbreviation for Matrine, Sophocarpine, and Pirfenidone, respectively.

Additionally, we found an interesting phenomenon that the anti-fibrotic potency was positively correlated with lipophilicity of the corresponding compounds, and a bubble chart based on calculated log partition coefficients (ClogP) and IC_50_ values was established. As depicted in Figure 3, compounds exhibiting better anti-fibrotic activities tended to possess higher ClogP values. The introduction of indoles significantly increased the ClogP values of matrine (from 1.36 to 1.98–4.18), which might permit better membrane penetration and consequently more potent activities. 

### 2.3. Inhibition Effects of Expression Levels of ECM Proteins by Key Compound ***3f***

**Figure 4:**
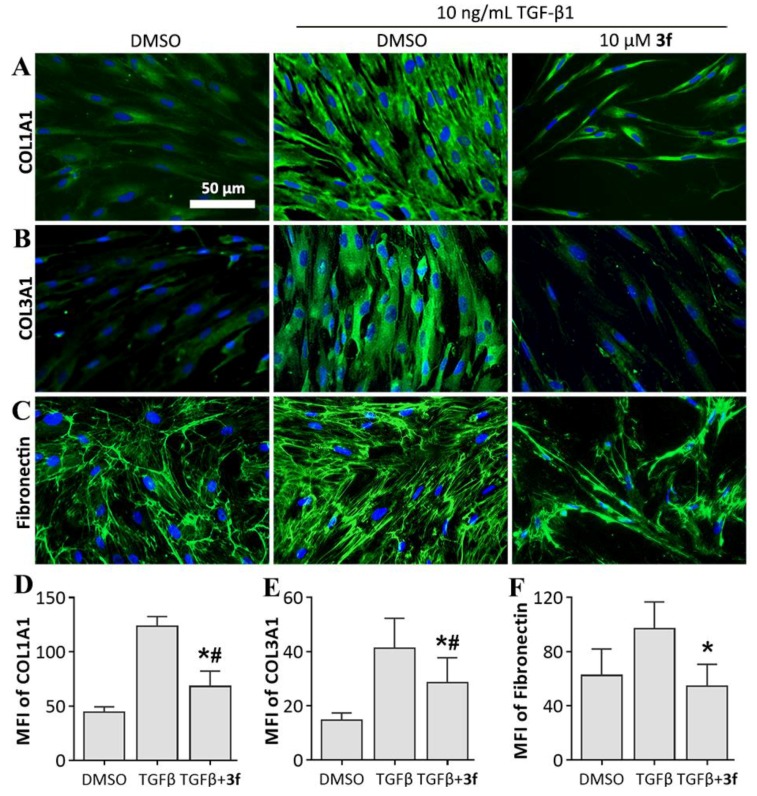
Effects of compound **3f** on inhibiting extracellular matrix (ECM) protein levels in MRC-5 cells. (**A**–**C**) Immunofluorescence staining of each protein; (**D**–**F**) Mean fluorescence intensity (MFI) of each protein, presented as mean ± SD of three individual experiments. * *p* < 0.05 vs TGFβ group, ^#^ *p* < 0.05 vs. DMSO group.

Due to the significance of collagen in IPF pathogenesis, we first evaluated the inhibitory effect of key compound **3f** against collagen accumulation. Immunofluorescence staining was employed to investigate collagen protein expression. As indicated in Figure 4A,B,D,E, collagen synthesis was significantly elevated upon TGF-β1 stimulation, and the expression of Collagen type I alpha-1 chain (COL1A1) and Collagen type III alpha-1 chain (COL3A1) was upregulated by 178.2% and 183.1%, respectively. 10 μM **3f** treatment resulted in remarkable decrease in expression level of COL1A1 and COL3A1 relative to TGF-β1 stimulation group (46.2% and 31.1%, respectively), indicating a potent inhibitory effect of **3f** against TGF-β1-induced collagen accumulation.

Next, we evaluated the inhibitory effect of **3f** against fibronectin expression. Fibronectin is another major constituent of ECM and plays a critical role in fibrogenesis by promoting the migration [22], proliferation [23], and adhesion [24] of fibroblasts. We observed that **3f** treatment reduced the expression level of fibronectin by 40.0% comparing to TGF-β1 stimulation group, which resulted in even lower expression level than vehicle group (Figure 4C,F). These results suggested that **3f** could revert the increase of ECM production induced by TGF-β1 stimulation. 

### 2.4. Inhibition Effects against Fibroblast-to-Myfibroblast Transition by ***3f***

**Figure 5:**
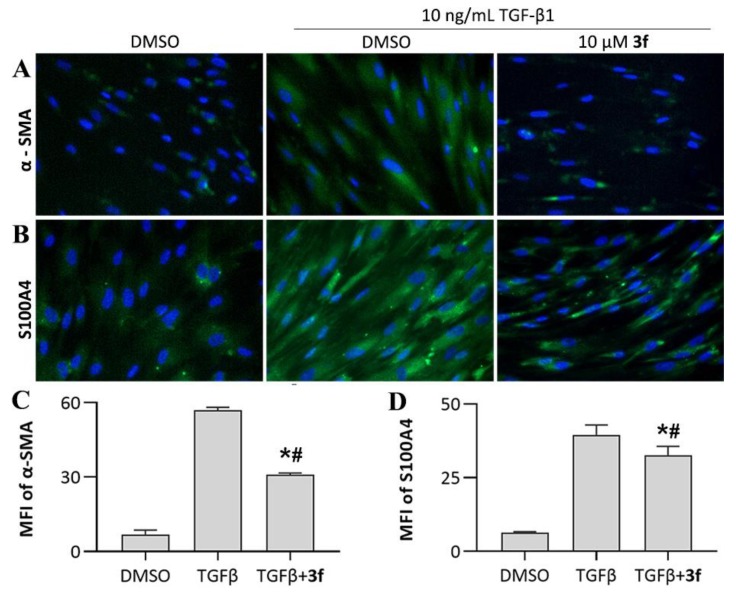
Effects of compound **3f** on inhibiting fibroblast-to-myofibroblast transition. (**A**,**B**) Immunofluorescence staining of myofibroblast markers; (**C**,**D**) Mean fluorescence intensity of myofibroblast markers, presented as mean ± SD of three individual experiments. * *p* < 0.05 vs. TGFβ group, ^#^ *p* < 0.05 vs. DMSO group.

Myofibroblasts, a further differentiated subset of fibroblasts, are primary effector cells for collagen synthesis in fibrotic disorders. Inhibition of fibroblast differentiation is an important therapeutic target for IPF treatment. To examine whether **3f** could inhibit TGF-β1-induced fibroblast-to-myofibroblast transition, we investigated the expression level of alpha-smooth muscle actin (α-SMA) and S100A4, the key markers which distinguish myofibroblasts from their precursor fibroblasts [25]. We observed dramatic upregulation of myofibroblast markers in response to TGF-β1 stimulation (774.9% and 537.7% for α-SMA and S100A4, respectively), characterizing a strong expression of myofibroblast phenotype. **3f** treatment resulted in disruption of fibroblast-to-myofibroblast transition, as evidenced by 45.8% and 17.7% downregulation in expression levels of α-SMA and S100A4, respectively, compared to TGF-β1 stimulation group (Figure 5). 

### 2.5. Inhibition Effects against Smad-Mediated Signaling by ***3f***

**Figure 6:**
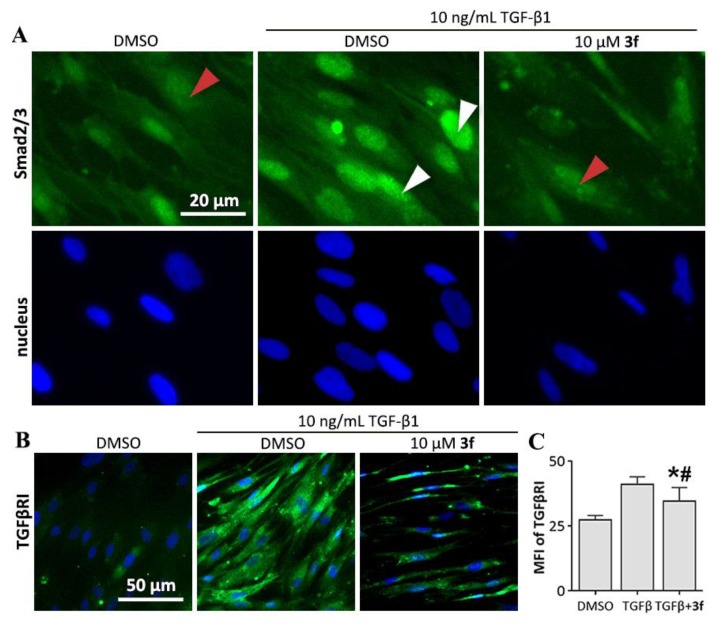
Effect of compound **3f** against small mothers against decapentaplegic homologs (Smad) signaling. (**A**) **3f** inhibited cytoplasm to nuclear translocation of Smad2/3 in MRC-5 cells. Immunofluorescence staining using an antibody recognizing total Smad2 and Smad3. Red arrows indicate the localization of Smad2/3 in cytoplasm, and white arrows indicate the nuclear localization of Smad2/3; (**B**) **3f** inhibited TGF-β1-induced upregulation of expression level of TGF-β receptor type I (TGFβRI); (**C**) mean fluorescence intensity (MFI) of TGFβRI, presented as mean ± SD of three individual experiments. * *p* < 0.05 vs. TGFβ group, ^#^ *p* < 0.05 vs. DMSO group.

Small mothers against decapentaplegic homologs (Smad) are key mediators and regulators of TGFβ signaling. The binding of TGF-β1 to TGFβ receptors leads to the phosphorylation of Smad2/3 complex, which then translocate into nucleus to enhance gene transcription by cooperating with DNA transcription factors [26]. Inhibition of the nuclear translocation of Smad2/3 could attenuate the transcription of profibrotic genes such as collagens and α-SMA. Therefore, we investigated the cellular localization of Smad2/3 by immunofluorescence assay. As depicted in Figure 6A, Smad2/3 predominately localize in cytoplasm under normal conditions, but translocate into nucleus upon TGF-β1 stimulation. We observed that **3f** could significantly inhibit the cytoplasm-to-nuclear translocation of Smad2/3.

Another emerging effect of Smad which acts as the amplifier of TGF-β signaling also contribute to fibrotic diseases. The nuclear translocation of Smad3 leads to the activation of the miR-433 promotor region. miR-433 promotes the decrease of antizyme inhibitor I (Azin1) and eventually results in depletion of polyamine, which causes the upregulation of expression level of TGF-β receptor type I (TGFβRI) and TGF-β1. This series of signaling creates a positive-feedback loop which amplify the TGFβ signaling [27]. TGF-β1-induced upregulation of expression level of TGFβRI was also observed in this study (Figure 6B), which was reduced by 46.7% under **3f** treatment (Figure 6C), suggesting an inhibitory effect of **3f** against the positive-feedback loop of TGFβ signaling.

### 2.6. Inhibition Effects against TGF-β1 Induced Migration of MRC-5 Cells by ***3f***

**Figure 7:**
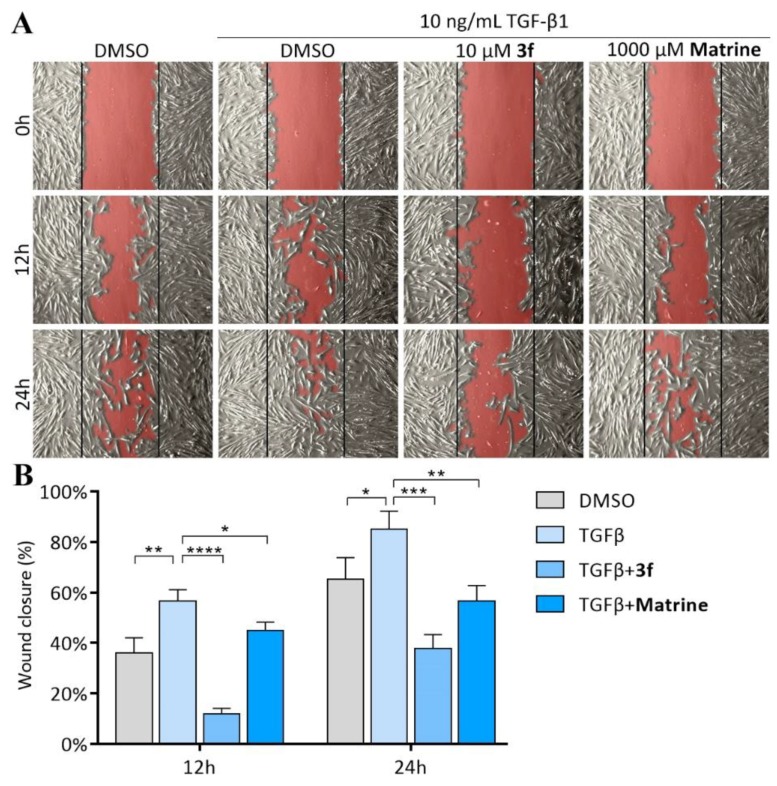
Effects of compound **3f** and matrine on inhibiting TGF-β1-induced MRC-5 fibroblasts migration. (**A**) Microscopic picture taken on 0 h, 12 h, and 24 h, the open area of wound region was highlighted in red; (**B**) Percentage of closure of wound area, analyzed by ImageJ software and MRI would healing tool plugin. Data are presented as mean ± SD of three individual experiments. * *p* < 0.05, ** *p* < 0.01, *** *p* < 0.001, **** *p* < 0.0001 vs. TGFβ group.

Fibroblast migration is the key event in pulmonary fibrogenesis. Lung fibroblasts migrate across basement membranes and deposit excessive collagen which resulted in loss of function and eventually destruction of alveoli [28]. Inhibition of lung fibroblasts migration is a promising potential therapeutic target for IPF. We performed wound healing assay to investigate the inhibitory effect of **3f** and matrine against fibroblast migration (Figure 7). The results indicated that MRC-5 fibroblasts displayed limited migration capacity under normal conditions, with 65.2% closure of wound area over 24 h period. However, wound closure increased to 85.0% over 24 h period upon TGF-β1 stimulation, indicating a significant enhancement in migration capacity, whilst treatment of **3f** resulted in potent inhibition of TGF-β1-induced fibroblast migration, with only 37.7% closure of wound area over 24 h period. It is also worth mentioning that the inhibitory effect of 10 μM **3f** was more potent than that of 1000 μM matrine (56.6% closure of wound area over 24 h period). 

### 2.7. Action on TGFβ/Smad Pathway of ***3f***

**Figure 8:**
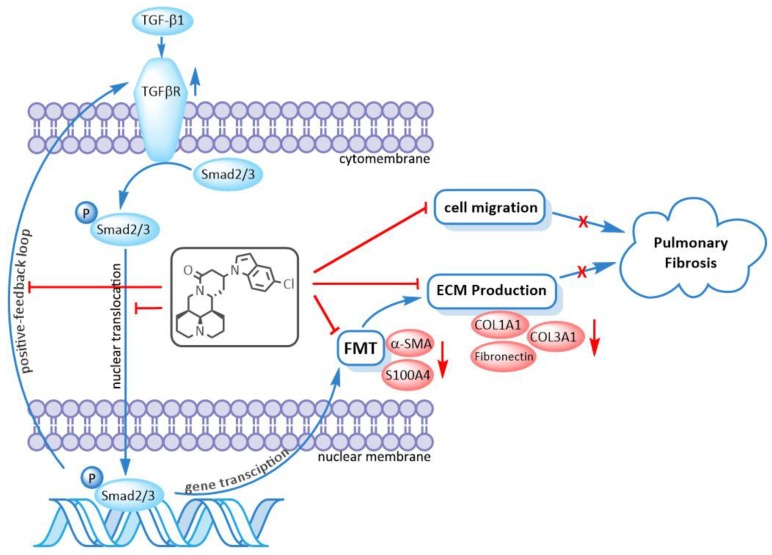
Compound **3f** exerted anti-pulmonary fibrosis activity via repression of the TGFβ/Smad signaling pathway. FMT is abbreviation for fibroblast-to-myofibroblast transition.

As illustrated earlier, **3f** could significantly inhibit fibroblast-to-myofibroblast transition induced by TGF-β1, which led to the downregulation of ECM production. **3f** could also inhibit Smad signaling by inhibiting cytoplasm-to-nuclear translocation of Smad2/3. The inhibition of positive-feedback loop of TGFβ signaling was evidenced by suppression of TGF-β1-induced upregulation of TGFβRI by **3f** as well. Therefore, it was speculated that **3f** might exert anti-pulmonary fibrosis activity via repression of the TGF-β/Smad signaling pathway, as depicted in Figure 8.

## 3. Experimental Section

### 3.1. Apparatus, Materials, and Analysis Reagents

Matrine (98%) and sophocarpine (95%) were obtained from Yanchi Dushun biotechnology limited company (Ningxia, China). Pirfenidone (98%) were obtained from TCI development limited company (Shanghai, China). All reagents were used without further purification as received from commercial sources. Reactions were monitored by Thin Layer Chromatography on plates (GF_254_) supplied by Yantai Chemicals (China). If not specially mentioned, flash column chromatography was performed on silica gel (200–300 mesh) supplied by Tsingtao Haiyang Chemicals (China).^1^H-, ^13^C-NMR, and NOE spectra were taken in Methanol-*d*_4_ or CDCl_3_ at room temperature on Bruker Avance III 600 MHz instruments (Bruker, Rheinstetten, Germany) using the solvent signals (Methanol-*d*_4_: *δ*_H_ 3.31 ppm/*δ*_C_ 49.0 ppm; CDCl_3_: *δ*_H_ 7.26 ppm/*δ*_C_ 77.4 ppm) as references. ESI high-resolution mass spectra (ESI-HRMS) data were acquired using Q-TOF analyzer in SYNAPT HDMS system (Waters, Milford, MA, USA). Microwave irradiation experiments were carried out in a Discover SP microwave synthesizer (CEM, Matthews, NC, USA).

### 3.2. Chemistry

#### 3.2.1. General Procedure for the Synthesis of Compounds **2a**–**d**

To a 10 mL microwave vial was added sophocarpine (0.3 mmol) and aliphatic amine (1.5 mmol), then water (1 mL) was added as solvent. The solution was irradiated under the microwave power of 100 W for 30 min. After irradiation, the reaction solution was poured into saturated aqueous ammonium chloride (20 mL), and then extracted with dichloromethane (20 mL) twice. The organic layer was washed by saturated sodium chloride, dried over anhydrous sodium sulfate, and concentrated under reduced pressure. The residue was purified by flash chromatography on silica gel with dichloromethane/methanol as the eluent. Compounds **2a**–**d** were mostly presented in pairs of 13*S*/13*R* stereoisomers, of which 13*S*-isomers were the major stereoisomers. The NMR peaks recorded below were signals of the major stereoisomers.

*13-methylamino-matrine* (**2a**). Sophocarpine (73.9 mg, 0.3 mmol) was treated with methylamine (1.5 mmol) according to the general procedure, then purified by silica gel column chromatography with dichloromethane/methanol as the eluents to give the desired product **2a** as a light-yellow solid, yield: 95.2%; ^1^H-NMR (600 MHz, MeOD-*d*_4_) δ 4.25 (dd, *J* = 12.7, 4.4 Hz, 1H), 3.98 (dt, *J* = 11.7, 6.2 Hz, 1H), 3.13 (t, *J* = 12.7 Hz, 1H), 2.95–2.92 (m, 1H), 2.85 (d, *J* = 11.5 Hz, 1H), 2.81 (d, *J* = 11.5 Hz, 1H), 2.57 (dd, *J* = 17.1, 4.6 Hz, 1H), 2.38 (s, 3H), 2.27 (dd, *J* = 17.2, 6.5 Hz, 1H), 2.21 (brs, 1H), 2.05–1.93 (m, 4H), 1.89 (ddd, *J* = 13.8, 6.6, 2.8 Hz, 1H), 1.78–1.57 (m, 6H), 1.49–1.44 (m, 3H). ^13^C-NMR (150 MHz, MeOD-*d*_4_) δ 169.93, 65.21, 58.33, 58.26, 52.01, 51.06, 43.54, 43.21, 38.44, 37.16, 33.43, 30.69, 28.81, 27.46, 22.15, 21.64; ESI-HRMS *m*/*z* calcd for C_16_H_28_N_3_O [M + H]^+^, 278.2227; found 278.2216. 

*13-dimethylamino-matrine* (**2b**). Sophocarpine (73.9 mg, 0.3 mmol) was treated with dimethylamine (1.5 mmol) according to the general procedure, then purified by silica gel column chromatography with dichloromethane/methanol as the eluents to give the desired product **2b** as a white solid, yield: 93.8%; ^1^H-NMR (600 MHz, MeOD-*d*_4_) δ 4.23 (dd, *J* = 12.6, 4.4 Hz, 1H), 4.04 (dt, *J* = 11.0, 5.5 Hz, 1H), 3.13 (t, *J* = 12.7 Hz, 1H), 2.87 (d, *J* = 11.3 Hz, 1H), 2.82 (d, *J* = 11.4 Hz, 1H), 2.69–2.64 (m, 1H), 2.53 (dd, *J* = 17.0, 3.6 Hz, 1H), 2.41 (dd, *J* = 16.9, 8.4 Hz, 1H), 2.31 (s, 6H), 2.26 (brs, 1H), 2.07–1.95 (m, 5H), 1.78–1.57 (m, 6H), 1.49–1.45 (m, 3H). ^13^C-NMR (150 MHz, MeOD-*d*_4_) δ 169.86, 65.38, 58.29, 58.23, 56.39, 52.57, 43.56, 42.66, 42.27 (2), 37.33, 36.24, 28.70, 28.31, 27.49, 22.12, 21.66; ESI-HRMS *m*/*z* calcd for C_17_H_30_N_3_O [M + H]^+^, 292.2383; found 292.2371. 

*13-(pyrrolidine-1-yl)-matrine* (**2c**). Sophocarpine (73.9 mg, 0.3 mmol) was treated with pyrrolidine (1.5 mmol) according to the general procedure, then purified by silica gel column chromatography with dichloromethane/methanol as the eluents to give the desired product **2c** as a light-yellow solid, yield: 93.1%; ^1^H-NMR (600 MHz, MeOD-*d*_4_) δ 4.32 (dd, *J* = 12.7, 4.5 Hz, 1H), 4.01 (dt, *J* = 11.3, 5.8 Hz, 1H), 3.09 (t, *J* = 13.0 Hz, 1H), 2.86–2.79 (m, 2H), 2.64–2.55 (m, 5H), 2.50–2.38 (m, 2H), 2.14 (brs, 1H), 2.00–1.87 (m, 4H), 1.83–1.39 (m, 14H); ^13^C-NMR (150 MHz, MeOD-*d*_4_) δ 167.80, 64.08, 57.28, 57.23, 55.48, 51.92 (2), 50.84, 42.00, 41.87, 38.54, 35.74, 30.32, 27.70, 26.76, 23.36 (2), 21.18, 20.78; ESI-HRMS *m*/*z* calcd for C_19_H_32_N_3_O [M + H]^+^, 318.2540; found 318.2549. 

*13-morpholinocyclohexane-matrine* (**2d**). Sophocarpine (73.9 mg, 0.3 mmol) was treated with morpholine (1.5 mmol) according to the general procedure, then purified by silica gel column chromatography with dichloromethane/methanol as the eluents to give the desired product **2d** as a white solid, yield: 94.5%; ^1^H-NMR (600 MHz, MeOD-*d*_4_) δ 4.31–4.25 (m, 1H), 4.00 (dt, *J* = 11.9, 6.1 Hz, 1H), 3.70 (brs, 4H), 3.11–3.04 (m, 1H), 2.89 (brs, 2H), 2.70–1.93 (m, 13H), 1.78–1.50 (m, 9H); ^13^C-NMR (150 MHz, MeOD-*d*_4_) δ 170.16, 67.94 (2), 65.30, 58.19, 58.12, 56.00, 51.45 (2), 50.54, 43.16, 42.86, 37.02, 36.20, 31.63, 28.36, 27.26, 21.99, 21.58; ESI-HRMS *m*/*z* calcd for C_19_H_32_N_3_O_2_ [M + H]^+^, 334.2489; found 334.2493. 

#### 3.2.2. General Procedure for the Synthesis of Compounds **3a**–**n**


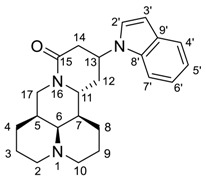


To a solution of the corresponding substituted indole (0.36 mmol) in THF (2 mL) was added 2 mol/L KHDMS (0.36 mmol) at −20 °C; the resulting solution was stirred at the same temperature for 20 min, and then sophocarpine (0.3 mmol) was added. The reaction temperature was allowed to raise to r.t. and stirred overnight. After the completion of the reaction, the reaction mixture was diluted with dichloromethane (50 mL). The organic layer was washed with water and brine, dried over anhydrous sodium sulfate, and concentrated under reduced pressure. The residue was purified by flash chromatography on silica gel with dichloromethane/methanol as the eluent. Compounds **3a**–**n** were mostly presented in pairs of 13*S*/13*R* stereoisomers, of which 13*S*-isomers were the major stereoisomers. The NMR peaks recorded below were signals of the major stereoisomers. 

*13-(1H-indol-1-yl)-matrine* (**3a**). Sophocarpine (73.9 mg, 0.3 mmol) was treated with indole (42.2 mg, 0.36 mmol) according to the general procedure, then purified by silica gel column chromatography with dichloromethane/methanol as the eluents to give the desired product **3a** as a white solid, yield: 65.1%; ^1^H-NMR (600 MHz, MeOD-*d*_4_) δ 7.56 (d, *J* = 7.8 Hz, 1H, 4’-CH), 7.45 (d, *J* = 8.3 Hz, 1H, 7’-CH), 7.18–7.16 (m, 2H, 6’-CH, 3’-CH), 7.05 (t, *J* = 7.4 Hz, 1H, 5’-CH), 6.50 (d, *J* = 3.3 Hz, 1H, 2’-CH), 4.97–4.93 (m, 1H, 13-CH), 4.35 (dd, *J* = 12.7, 4.4 Hz, 1H, 17α-CH_2_), 3.85 (dt, *J* = 11.4, 5.8 Hz, 1H, 11-CH), 3.13 (t, *J* = 12.7 Hz, 1H, 17β-CH_2_), 2.97 (dd, *J* = 17.3, 5.4 Hz, 1H, 14α-CH_2_), 2.87–2.74 (m, 3H, 14β-CH_2_, 10α-CH_2_, 2α-CH_2_), 2.53–2.48 (m, 1H, 12α-CH_2_), 2.24 (brs, 1H, 6-CH), 2.15–2.12 (m, 1H, 12β-CH_2_), 2.04–1.95 (m, 2H, 2β-CH_2_, 10β-CH_2_), 1.91–1.31 (m, 10H, 5-CH, 7-CH, 3-CH_2_, 4-CH_2_, 8-CH_2_, 9-CH_2_); ^13^C-NMR (151 MHz, MeOD-*d*_4_) δ 169.48 (15-C), 137.16 (8’-C), 130.17 (9’-C), 124.77 (2’-CH), 122.67 (6’-CH), 121.93 (4’-CH), 120.66 (5’-CH), 110.31 (7’-CH), 103.14 (3’-CH), 65.14 (6-CH), 58.28 (2-CH_2_, 10-CH_2_), 52.44 (11-CH), 47.56 (13-CH), 43.44 (17-CH_2_), 42.82 (7-CH), 38.66 (14-CH_2_), 37.07 (5-CH), 31.48 (12-CH_2_), 28.71 (4-CH_2_), 27.44 (8-CH_2_), 22.08 (3-CH_2_), 21.42 (9-CH_2_); ESI-HRMS *m*/*z* Calcd for C_23_H_30_N_3_O [M + H]^+^, 364.2383; found 364.2382. 

*13-(3-methyl-1H-indol-1-yl)-matrine* (**3b**). Sophocarpine (73.9 mg, 0.3 mmol) was treated with 3-methylindole (47.2 mg, 0.36 mmol) according to the general procedure, then purified by silica gel column chromatography with dichloromethane/methanol as the eluents to give the desired product **3b** as a white solid, yield: 35.2%; ^1^H-NMR (600 MHz, MeOD-*d*_4_) δ 7.50 (d, *J* = 7.9 Hz, 1H, 4’-CH), 7.38 (d, *J* = 8.4 Hz, 1H, 7’-CH), 7.16 (t, *J* = 8.2 Hz, 1H, 6’-CH), 7.04 (t, *J* = 7.9 Hz, 1H, 5’-CH), 6.92 (d, *J* = 1.1 Hz, 1H, 2’-CH), 4.90–4.86 (m, 1H, 13-CH), 4.35 (dd, *J* = 12.7, 4.8 Hz, 1H, 17α-CH_2_), 3.81 (dt, *J* = 11.7, 6.0 Hz, 1H, 11-CH), 3.10 (t, *J* = 12.7 Hz, 1H, 17β-CH_2_), 2.94–2.73 (m, 4H, 14-CH_2_, 10α-CH_2_, 2α-CH_2_), 2.45 (ddd, *J* = 14.1, 8.1, 5.5 Hz, 1H, 12α-CH_2_), 2.29 (d, *J* = 1.02 Hz, 3H, Ph-Me), 2.28–2.19 (m, 1H, 6-CH), 2.09–1.87 (m, 4H, 12β-CH_2_, 2β-CH_2_, 10β-CH_2_, 5-CH), 1.82–1.29 (m, 9H, 7-CH, 3-CH_2_, 4-CH_2_, 8-CH_2_, 9-CH_2_); ^13^C-NMR (151 MHz, MeOD-*d*_4_) δ 169.63 (15-C), 137.51 (8’-C), 130.20 (9’-C), 122.68 (2’-CH), 122.38 (6’-CH), 120.04 (5’-CH), 119.91 (4’-CH), 112.21 (3’-C), 110.10 (7’-CH), 65.11 (6-CH), 58.11 (2-CH_2_, 10-CH_2_), 52.38 (11-CH), 47.24 (13-CH), 43.32 (17-CH_2_), 42.75 (7-CH), 38.64 (14-CH_2_), 36.96 (5-CH), 31.56 (12-CH_2_), 28.61 (4-CH_2_), 27.33 (8-CH_2_), 22.00 (3-CH_2_), 21.35 (9-CH_2_), 9.82 (Ph-CH_3_); ESI-HRMS *m*/*z* Calcd for C_24_H_32_N_3_O [M + H]^+^, 378.2540; found 378.2551. 

*13-(4-methoxy-1H-indol-1-yl)-matrine* (**3c**). Sophocarpine (73.9 mg, 0.3 mmol) was treated with 4-methoxyindole (53.0 mg, 0.36 mmol) according to the general procedure, then purified by silica gel column chromatography with dichloromethane/methanol as the eluents to give the desired product **3c** as a white solid, yield: 41.7%; ^1^H-NMR (600 MHz, MeOD-*d*_4_) δ 7.11–7.02 (m, 3H, 6’-CH, 7’-CH, 2’-CH), 6.57–6.52 (m, 2H, 5’-CH, 3’-CH), 4.34 (dd, *J* = 12.7, 4.4 Hz, 1H), 4.84–4.80 (m, 1H, 13-CH), 4.31 (dd, *J* = 12.6, 4.4 Hz, 1H, 17α-CH_2_), 3.90 (s, 3H, Ph-OMe), 3.80 (dt, *J* = 11.3, 5.8 Hz, 1H, 11-CH), 3.09 (t, *J* = 12.7 Hz, 1H, 17β-CH_2_), 2.91 (dd, *J* = 17.3, 5.4 Hz, 1H, 14α-CH_2_), 2.81–2.70 (m, 3H, 14β-CH_2_, 10α-CH_2_, 2α-CH_2_), 2.43–2.37 (m, 1H, 12α-CH_2_), 2.16 (brs, 1H, 6-CH), 2.06–2.04 (m, 1H, 12β-CH_2_), 1.99–1.96 (m, 2H, 2β-CH_2_, 10β-CH_2_), 1.83–1.79 (m, 1H, 5-CH), 1.74–1.26 (m, 9H, 7-CH, 3-CH_2_, 4-CH_2_, 8-CH_2_, 9-CH_2_); ^13^C-NMR (151 MHz, MeOD-*d*_4_) δ 169.31 (15-C), 154.86 (4’-C), 138.57 (8’-C), 123.73 (2’-CH), 123.12 (6’-CH), 120.49 (9’-C), 103.82 (5’-CH), 100.59 (3’-CH), 100.47 (7’-CH), 65.06 (6-CH), 58.21 (2-CH_2_), 58.11 (10-CH_2_), 55.64 (Ph-OMe), 52.39 (11-CH), 47.74 (13-CH), 43.42 (17-CH_2_), 42.65 (7-CH), 38.66 (14-CH_2_), 37.01 (5-CH), 31.39 (12-CH_2_), 28.67 (4-CH_2_), 27.41 (8-CH_2_), 22.05 (3-CH_2_), 21.37 (9-CH_2_); ESI-HRMS *m*/*z* Calcd for C_24_H_32_N_3_O_2_ [M + H]^+^, 394.2489; found 394.2481. 

*13-(4-nitro-1H-indol-1-yl)-matrine* (**3d**). Sophocarpine (73.9 mg, 0.3 mmol) was treated with 4-nitroindole (58.4 mg, 0.36 mmol) according to the general procedure, then purified by silica gel column chromatography with dichloromethane/methanol as the eluents to give the desired product **3d** as a yellow solid, yield: 68.3%; ^1^H-NMR (600 MHz, MeOD-*d*_4_) δ 8.09 (d, *J* = 8.0 Hz, 1H, 5’-CH), 7.96 (d, *J* = 8.2 Hz, 1H, 7’-CH), 7.56 (d, *J* = 3.3 Hz, 1H, 2’-CH), 7.33 (t, *J* = 8.1 Hz, 1H, 6’-CH), 7.19 (d, *J* = 3.2 Hz, 1H, 3’-CH), 5.09 (ddd, *J* = 9.2, 8.6, 4.2 Hz, 1H, 13-CH), 4.34 (dd, *J* = 12.7, 4.4 Hz, 1H, 17α-CH_2_), 3.94–3.90 (m, 1H, 11-CH), 3.14 (t, *J* = 12.7 Hz, 1H, 17β-CH_2_), 3.00 (dd, *J* = 17.3, 5.5 Hz, 1H, 14α-CH_2_), 2.87 (dd, *J* = 17.2, 7.7 Hz, 1H, 14β-CH_2_), 2.78–2.74 (m, 2H, 10α-CH_2_, 2α-CH_2_), 2.56–2.51 (m, 1H, 12α-CH_2_), 2.24–2.19 (m, 2H, 6-CH, 12β-CH_2_), 2.01–1.95 (m, 2H, 2β-CH_2_, 10β-CH_2_), 1.91–1.87 (m, 2H, 5-CH, 7-CH), 1.79–1.32 (m, 8H, 3-CH_2_, 4-CH_2_, 8-CH_2_, 9-CH_2_); ^13^C-NMR (151 MHz, MeOD-*d*_4_) δ 168.88 (15-C), 141.48 (4’-C), 139.40 (8’-C), 130.01 (2’-CH), 123.78 (9’-C), 121.74 (6’-CH), 118.50 (5’-CH), 117.79 (7’-CH), 103.53 (3’-CH), 65.17 (6-CH), 58.16 (2-CH_2_, 10-CH_2_), 52.51 (11-CH), 48.27 (13-CH), 43.57 (17-CH_2_), 42.55 (7-CH), 38.79 (14-CH_2_), 37.13 (5-CH), 31.26 (12-CH_2_), 28.67 (4-CH_2_), 27.45 (8-CH_2_), 22.05 (3-CH_2_), 21.40 (9-CH_2_); ESI-HRMS *m*/*z* Calcd for C_23_H_29_N_4_O [M + H]^+^, 409.2234; found 409.2222. 

*13-(5-methoxy-1H-indol-1-yl)-matrine* (**3e**). Sophocarpine (73.9 mg, 0.3 mmol) was treated with 5-methoxyindole (53.0 mg, 0.36 mmol) according to the general procedure, then purified by silica gel column chromatography with dichloromethane/methanol as the eluents to give the desired product **3e** as a white solid, yield: 55.1%; ^1^H-NMR (600 MHz, MeOD-*d*_4_) δ 7.31 (d, *J* = 8.8 Hz, 1H, 7’-CH), 7.11 (s, 1H, 2’-CH), 7.06 (s, 1H, 4’-CH), 6.82 (d, *J* = 7.5 Hz, 1H, 6’-CH), 6.41 (s, 1H, 3’-CH), 4.85–4.81 (m, 1H, 13-CH), 4.32 (d, *J* = 13.2 Hz, 1H, 17α-CH_2_), 3.82–3.80 (m, 1H, 11-CH), 3.79 (s, 3H, Ph-OMe), 3.10 (t, *J* = 12.9 Hz, 1H, 17β-CH_2_), 2.94–2.90 (m, 1H, 14α-CH_2_), 2.81–2.72 (m, 3H, 14β-CH_2_, 10α-CH_2_, 2α-CH_2_), 2.46–2.41 (m, 1H, 12α-CH_2_), 2.19 (brs, 1H, 6-CH), 2.08 (d, *J* = 13.3 Hz, 1H, 12β-CH_2_), 1.98–1.93 (m, 2H, 2β-CH_2_, 10β-CH_2_), 1.85 (d, *J* = 13.8 Hz, 1H, 5-CH), 1.78–1.28 (m, 9H, 7-CH, 3-CH_2_, 4-CH_2_, 8-CH_2_, 9-CH_2_); ^13^C-NMR (151 MHz, MeOD-*d*_4_) δ 169.44 (15-C), 155.61 (5’-C), 132.47 (9’-C), 130.61 (8’-C), 125.35 (2’-CH), 112.89 (6’-CH), 111.03 (4’-CH), 103.70 (7’-CH), 102.86 (3’-CH), 65.12 (6-CH), 58.18 (2-CH_2_, 10-CH_2_), 56.19 (Ph-OMe), 52.41 (11-CH), 47.70 (13-CH), 43.44 (17-CH_2_), 42.79 (7-CH), 38.71 (14-CH_2_), 37.07 (5-CH), 31.54 (12-CH_2_), 28.73 (4-CH_2_), 27.46 (8-CH_2_), 22.09 (3-CH_2_), 21.43 (9-CH_2_); ESI-HRMS *m*/*z* Calcd for C_24_H_32_N_3_O_2_ [M + H]^+^, 394.2489; found 394.2483. 

*13-(5-chloro-1H-indol-1-yl)-matrine* (**3f**). Sophocarpine (73.9 mg, 0.3 mmol) was treated with 5-chloroindole (54.6 mg, 0.36 mmol) according to the general procedure, then purified by silica gel column chromatography with dichloromethane/methanol as the eluents to give the desired product **3f** as a white solid, yield: 77.2%; ^1^H-NMR (600 MHz, MeOD-*d*_4_) δ 7.54 (d, *J* = 1.9 Hz, 1H, 4’-CH), 7.43 (d, *J* = 8.8 Hz, 1H, 7’-CH), 7.24 (d, *J* = 3.3 Hz, 1H, 2’-CH), 7.13 (d, *J* = 8.8, 2.0 Hz, 1H, 6’-CH), 6.48 (d, *J* = 3.0 Hz, 1H, 3’-CH), 4.94–4.90 (m, 1H, 13-CH), 4.33 (dd, *J* = 12.6, 4.4 Hz, 1H, 17α-CH_2_), 3.85 (dt, *J* = 11.3, 5.8 Hz, 1H, 11-CH), 3.12 (t, *J* = 12.7 Hz, 1H, 17β-CH_2_), 2.95 (dd, *J* = 16.9, 5.1 Hz, 1H, 14α-CH_2_), 2.82 (dd, *J* = 17.3, 7.3 Hz, 1H, 14β-CH_2_), 2.77–2.73 (m, 2H, 10α-CH_2_, 2α-CH_2_), 2.48 (ddd, *J* = 14.3, 8.9, 5.8 Hz, 1H, 12α-CH_2_), 2.22 (brs, 1H, 6-CH), 2.13 (dd, *J* = 13.8, 7.2 Hz, 1H, 12β-CH_2_), 2.00 –1.82 (m, 4H, 2β-CH_2_, 10β-CH_2_, 5-CH, 7-CH), 1.77–1.30 (m, 8H, 3-CH_2_, 4-CH_2_, 8-CH_2_, 9-CH_2_); ^13^C-NMR (151 MHz, MeOD-*d*_4_) δ 169.20 (15-C), 135.57 (8’-C), 131.16 (9’-C), 126.53 (2’-CH), 126.39 (5’-C), 122.78 (6’-CH), 121.18 (4’-CH), 111.65 (7’-CH), 102.89 (3-CH), 65.12 (6-CH), 58.16 (2-CH_2_,10-CH_2_), 52.42 (11-CH), 47.88 (13-CH), 43.46 (17-CH_2_), 42.72 (7-CH), 38.66 (14-CH_2_), 37.08 (5-CH), 31.37 (12-CH_2_), 28.69 (4-CH_2_), 27.44 (8-CH_2_), 22.07 (3-CH_2_), 21.41 (9-CH_2_); ESI-HRMS *m*/*z* Calcd for C_23_H_29_ClN_3_O [M + H]^+^, 398.1994; found 398.1999. 

*13-(5-bromo-1H-indol-1-yl)-matrine* (**3g**). Sophocarpine (73.9 mg, 0.3 mmol) was treated with 5-bromoindole (70.6 mg, 0.36 mmol) according to the general procedure, then purified by silica gel column chromatography with dichloromethane/methanol as the eluents to give the desired product **3g** as a white solid, yield: 62.1%; ^1^H-NMR (600 MHz, MeOD-*d*_4_) δ 7.70 (d, *J* = 1.9 Hz, 1H, 4’-CH), 7.39 (d, *J* = 8.9 Hz, 1H, 7’-CH), 7.25 (dd, *J* = 8.8, 1.9 Hz, 1H, 6’-CH), 7.22 (d, *J* = 3.3 Hz, 1H, 2’-CH), 6.48 (d, *J* = 3.3 Hz, 1H, 3’-CH), 4.94–4.90 (m, 1H, 13-CH), 4.32 (dd, *J* = 12.6, 4.4 Hz, 1H, 17α-CH_2_), 3.84 (dt, *J* = 11.3, 5.7 Hz, 1H, 11-CH), 3.11 (t, *J* = 12.7 Hz, 1H, 17β-CH_2_), 2.94 (dd, *J* = 17.3, 5.5 Hz, 1H, 14α-CH_2_), 2.84–2.72 (m, 3H, 14β-CH_2_, 10α-CH_2_, 2α-CH_2_), 2.46 (ddd, *J* = 15.1, 10.0, 6.1 Hz, 1H, 12α-CH_2_), 2.20 (brs, 1H, 6-CH), 2.14–2.10 (m, 1H, 12β-CH_2_), 2.04–1.93 (m, 2H, 2β-CH_2_, 10β-CH_2_), 1.85 (d, *J* = 14.4 Hz, 1H, 5-CH), 1.82–1.29 (m, 9H, 7-CH, 3-CH_2_, 4-CH_2_, 8-CH_2_, 9-CH_2_); ^13^C-NMR (151 MHz, MeOD-*d*_4_) δ 169.17 (15-C), 135.84 (8’-C), 131.84 (9’-C), 126.42 (2’-CH), 125.41 (6’-CH), 124.37 (4’-CH), 113.85 (5’-C), 112.12 (7’-CH), 102.85 (3’-CH), 65.13 (6-CH), 58.17 (2-CH_2_, 10-CH_2_), 52.43 (11-CH), 47.88 (13-CH), 43.49 (17-CH_2_), 42.72 (7-CH), 38.67 (14-CH_2_), 37.10 (5-CH), 31.36 (12-CH_2_), 28.71 (4-CH_2_), 27.47 (8-CH_2_), 22.11 (3-CH_2_), 21.44 (9-CH_2_); ESI-HRMS *m*/*z* Calcd for C_23_H_29_BrN_3_O [M + H]^+^, 442.1489; found 442.1474. 

*13-(5-cyano-1H-indol-1-yl)-matrine(***3h**). Sophocarpine (73.9 mg, 0.3 mmol) was treated with indole-5-carbonitrile (51.2 mg, 0.36 mmol) according to the general procedure, then purified by silica gel column chromatography with dichloromethane/methanol as the eluents to give the desired product **3h** as a white solid, yield: 68.9%; ^1^H-NMR (600 MHz, MeOD-*d*_4_) δ 8.01 (d, *J* = 1.6 Hz, 1H, 4’-CH), 7.66 (d, *J* = 8.6 Hz, 1H, 7’-CH), 7.45 (dd, *J* = 8.5, 1.6 Hz, 1H, 6’-CH), 7.43 (d, *J* = 3.4 Hz, 1H, 2’-CH), 6.68 (d, *J* = 3.4 Hz, 1H, 3’-CH), 5.04 (tdd, *J* = 8.6, 5.4, 2.9 Hz, 1H, 13-CH), 4.34 (dd, *J* = 12.6, 4.4 Hz, 1H, 17α-CH_2_), 3.91 (dt, *J* = 11.1, 5.6 Hz, 1H, 11-CH), 3.14 (t, *J* = 12.7 Hz, 1H, 17β-CH_2_), 2.97 (dd, *J* = 17.3, 5.6 Hz, 1H, 14α-CH_2_), 2.87–2.76 (m, 3H, 14β-CH_2_, 10α-CH_2_, 2α-CH_2_), 2.54–2.49 (m, 1H, 12α-CH_2_), 2.25 (brs, 1H, 6-CH), 2.22–2.18 (m, 1H, 12β-CH_2_), 2.06–1.96 (m, 2H, 2β-CH_2_, 10β-CH_2_), 1.91–1.88 (m, 1H, 5-CH), 1.80–1.32 (m, 9H, 7-CH, 3-CH_2_, 4-CH_2_, 8-CH_2_, 9-CH_2_); ^13^C-NMR (151 MHz, MeOD-*d*_4_) δ 168.93 (15-C), 138.76 (8’-C), 129.87 (9’-C), 127.82 (2’-CH), 127.67 (4’-CH), 125.46 (6’-CH), 121.50 (Ph-CN), 111.79 (7’-CH), 104.36 (3’-CH), 103.52 (5’-CH), 65.15 (6-CH), 58.24 (2-CH_2_, 10-CH_2_), 52.50 (11-CH), 48.05 (13-CH), 43.54 (17-CH_2_), 42.55 (7-CH), 38.67 (14-CH_2_), 37.11 (5-CH), 31.19 (12-CH_2_), 28.66 (4-CH_2_), 27.44 (8-CH_2_), 22.05 (3-CH_2_), 21.40 (9-CH_2_); ESI-HRMS *m*/*z* Calcd for C_24_H_29_N_4_O [M + H]^+^, 389.2336; found 389.2345. 

*13-(5-nitro-1H-indol-1-yl)-matrine* (**3i**). Sophocarpine (73.9 mg, 0.3 mmol) was treated with 5-nitroindole (58.4 mg, 0.36 mmol) according to the general procedure, then purified by silica gel column chromatography with dichloromethane/methanol as the eluents to give the desired product **3i** as a yellow solid, yield: 68.0%; ^1^H-NMR (600 MHz, MeOD-*d*_4_) δ 8.55 (d, *J* = 2.2 Hz, 1H, 4’-CH), 8.09 (dd, *J* = 9.1, 2.2 Hz, 1H, 6’-CH), 7.64 (d, *J* = 9.1 Hz, 1H, 7’-CH), 7.47 (d, *J* = 3.4 Hz, 1H, 2’-CH), 6.78 (d, *J* = 3.3 Hz, 1H, 3’-CH), 5.07 (tdd, *J* = 8.6, 5.4, 3.0 Hz, 1H, 13-CH), 4.34 (dd, *J* = 12.7, 4.4 Hz, 1H, 17α-CH_2_), 3.94 (dt, *J* = 11.1, 5.6 Hz, 1H, 11-CH), 3.15 (t, *J* = 12.6 Hz, 1H, 17β-CH_2_), 2.98 (dd, *J* = 17.4, 5.4 Hz, 1H, 14α-CH_2_), 2.89–2.77 (m, 3H, 14β-CH_2_, 10α-CH_2_, 2α-CH_2_), 2.54 (ddd, *J* = 14.5, 9.3, 5.7 Hz, 1H, 12α-CH_2_), 2.26–2.21 (m, 2H, 6-CH, 12β-CH_2_), 2.05–1.89 (m, 3H, 2β-CH_2_, 10β-CH_2_, 5-CH), 1.81–1.33 (m, 9H, 7-CH, 3-CH_2_, 4-CH_2_, 8-CH_2_, 9-CH_2_); ^13^C-NMR (151 MHz, MeOD-*d*_4_) δ 168.91 (15-C), 143.07 (8’-C), 139.97 (9’-C), 129.31 (5’-C), 128.79 (2’-CH), 118.89 (6’-CH), 118.04 (4’-CH), 110.77 (7’-CH), 105.91 (3’-CH), 65.22 (6-CH), 58.22 (2-CH_2_, 10-CH_2_), 52.57 (11-CH), 48.41 (13-CH), 43.62 (17-CH_2_), 42.63 (7-CH), 38.71 (14-CH_2_), 37.19 (5-CH), 31.23 (12-CH_2_), 28.73 (4-CH_2_), 27.51 (8-CH_2_), 22.11 (3-CH_2_), 21.46 (9-CH_2_); ESI-HRMS *m*/*z* Calcd for C_23_H_29_N_4_O [M + H]^+^, 409.2234; found 409.2221. 

*13-(1H-pyrrolo[3,2-c]pyridin-1-yl)-matrine* (**3j**). Sophocarpine (73.9 mg, 0.3 mmol) was treated with 5-azaindole (42.5 mg, 0.36 mmol) according to the general procedure, then purified by silica gel column chromatography with dichloromethane/methanol as the eluents to give the desired product **3j** as a yellow solid, yield: 37.6%; ^1^H-NMR (600 MHz, MeOD-*d*_4_) δ 8.80 (s, 1H, 4’-CH), 8.20 (d, *J* = 6.0 Hz, 1H, 6’-CH), 7.58 (d, *J* = 6.0 Hz, 1H, 7’-CH), 7.40 (d, *J* = 3.3 Hz, 1H, 2’-CH), 6.74 (d, *J* = 3.1 Hz, 1H, 3’-CH), 5.06–5.01 (m, 1H, 11-CH), 4.34 (dd, *J* = 12.6, 4.1 Hz, 1H, 17α-CH_2_), 3.94 (dt, *J* = 10.9, 5.4 Hz, 1H, 11-CH), 3.15 (t, *J* = 12.6 Hz, 1H, 17β-CH_2_), 2.97 (dd, *J* = 17.3, 5.4 Hz, 1H, 14α-CH_2_), 2.88 (dd, *J* = 17.3, 7.7 Hz, 1H, 14β-CH_2_), 2.77 (d, *J* = 11.3 Hz, 2H, 10α-CH_2_, 2α-CH_2_), 2.54 (tt, *J* = 9.2, 5.6 Hz, 1H, 12α-CH_2_), 2.25 (brs, 1H, 6-CH), 2.21 (d, *J* = 15.0 Hz, 1H, 12β-CH_2_), 2.00–1.97 (m, 2H, 2β-CH_2_, 10β-CH_2_), 1.92– 1.88 (m, 2H, 5-CH, 7-CH), 1.80 –1.33 (m, 8H, 3-CH_2_, 4-CH_2_, 8-CH_2_, 9-CH_2_); ^13^C-NMR (151 MHz, MeOD-*d*_4_) δ 168.85 (15-C), 144.09 (4’-CH), 140.98 (8’-C), 140.52 (6’-CH), 127.37 (2’-CH), 127.01 (9’-C), 106.61 (7’-CH), 103.23 (3’-CH), 65.17 (6-CH), 58.18 (2-CH_2_, 10-CH_2_), 52.52 (11-CH), 48.14 (13-CH), 43.58 (17-CH_2_), 42.59 (7-CH), 38.63 (14-CH_2_), 37.16 (5-CH), 31.18 (12-CH_2_), 28.70 (4-CH_2_), 27.47 (8-CH_2_), 22.08 (3-CH_2_), 21.43 (9-CH_2_); ESI-HRMS *m*/*z* Calcd for C_24_H_29_N_4_O [M + H]^+^, 365.2336; found 365.2330. 

*13-(6-fluoro-1H-indol-1-yl)-matrine* (**3k**). Sophocarpine (73.9 mg, 0.3 mmol) was treated with 6-fluoroindole (48.7 mg, 0.36 mmol) according to the general procedure, then purified by silica gel column chromatography with dichloromethane/methanol as the eluents to give the desired product **3k** as a yellow solid, yield: 69.1%; ^1^H-NMR (600 MHz, MeOD-*d*_4_) δ 7.50 (dd, *J* = 8.8, 5.4 Hz, 1H, 4’-CH), 7.22 (dd, *J* = 10.3, 2.1 Hz, 1H, 5’-CH), 7.17 (d, *J* = 3.4 Hz, 1H, 2’-CH), 6.84 (td, *J* = 8.7, 2.2 Hz, 1H, 7’-CH), 6.50 (t, *J* = 3.1 Hz, 1H, 3’-CH), 4.88–4.84 (m, 1H, 13-CH), 4.33 (dd, *J* = 14.1, 3.3 Hz, 1H, 17α-CH_2_), 3.85 (dt, *J* = 11.3, 5.8 Hz, 1H, 11-CH), 3.12 (t, *J* = 12.7 Hz, 1H, 17β-CH_2_), 2.94 (dd, *J* = 18.3, 5.4 Hz, 1H, 14α-CH_2_), 2.86–2.73 (m, 3H, 14β-CH_2_, 10α-CH_2_, 2α-CH_2_), 2.51–2.44 (m, 1H, 12α-CH_2_), 2.20 (brs, 1H, 6-CH), 2.12–1.91 (m, 3H, 12β-CH_2_, 2β-CH_2_, 10β-CH_2_), 1.88–1.30 (m, 10H, 5-CH, 7-CH, 3-CH_2_, 4-CH_2_, 8-CH_2_, 9-CH_2_); ^13^C-NMR (151 MHz, MeOD-*d*_4_) δ 169.26 (15-C), 161.24 (d, *J* = 236.2 Hz, 6’-C), 137.16 (d, *J* = 12.1 Hz, 8’-C), 126.59 (2’-CH), 125.53 (d, *J* = 3.7 Hz, 9’-C), 122.76 (d, *J* = 10.1 Hz, 4’-CH), 109.14 (d, *J* = 24.7 Hz, 7’-CH), 103.48 (3’-CH), 96.78 (d, *J* = 27.0 Hz, 5’-CH), 65.13 (6-CH), 58.19 (10-CH_2_), 58.17 (10-CH_2_), 52.43 (11-CH), 47.81 (13-CH), 43.47 (17-CH_2_), 42.72 (7-CH), 38.63 (14-CH_2_), 37.07 (5-CH), 31.28 (12-CH_2_), 28.73 (4-CH_2_), 27.44 (8-CH_2_), 22.10 (3-CH_2_), 21.43 (9-CH_2_); ESI-HRMS *m*/*z* Calcd for C_23_H_29_N_3_FO [M + H]^+^, 382.2289; found 382.2276. 

*13-(6-chloro-1H-indol-1-yl)-matrine* (**3l**). Sophocarpine (73.9 mg, 0.3 mmol) was treated with 6-chloroindole (54.6 mg, 0.36 mmol) according to the general procedure, then purified by silica gel column chromatography with dichloromethane/methanol as the eluents to give the desired product **3l** as a white solid, yield: 64.2%; ^1^H-NMR (600 MHz, MeOD-*d*_4_) δ 7.54–7.49 (m, 2H, 4’-CH, 5’-CH), 7.21 (d, *J* = 3.3 Hz, 1H, 2’-CH), 7.03 (d, *J* = 9.1 Hz, 1H, 7’-CH), 6.51 (d, *J* = 6.5 Hz, 1H, 3’-CH), 4.94–4.91 (m, 1H, 13-CH), 4.34 (dd, *J* = 12.8, 4.4 Hz, 1H, 17α-CH_2_), 3.86 (dt, *J* = 11.3, 5.6 Hz, 1H, 11-CH), 3.12 (t, *J* = 12.7 Hz, 1H, 17β-CH_2_), 2.94 (dd, *J* = 17.3, 5.4 Hz, 1H, 14α-CH_2_), 2.87–2.76 (m, 3H, 14β-CH_2_, 10α-CH_2_, 2α-CH_2_), 2.49–2.44 (m, 1H, 12α-CH_2_), 2.24 (brs, 1H, 6-CH), 2.15–2.12 (m, 1H, 12β-CH_2_), 2.01–1.96 (m, 2H2β-CH_2_, 10β-CH_2_), 1.90–1.83 (m, 2H, 5-CH, 7-CH), 1.79–1.33 (m, 8H, 3-CH_2_, 4-CH_2_, 8-CH_2_, 9-CH_2_); ^13^C-NMR (151 MHz, MeOD-*d*_4_) δ 169.24 (15-C), 137.58 (8’-C), 128.72 (6’-C), 128.68 (9’-C), 125.87 (2’-CH), 122.94 (4’-CH), 121.24 (5’-CH), 110.50 (7’-CH), 103.48 (3’-CH), 65.13 (6-CH), 58.16 (2-CH_2_, 10-CH_2_), 52.45 (11-CH), 47.72 (13-CH), 43.47 (17-CH_2_), 42.68 (7-CH), 38.65 (14-CH_2_), 37.04 (5-CH), 31.32 (12-CH_2_), 28.68 (4-CH_2_), 27.42 (8-CH_2_), 22.06 (3-CH_2_), 21.42 (9-CH_2_); ESI-HRMS *m*/*z* Calcd for C_23_H_29_ClN_3_O [M + H]^+^, 398.1994; found 398.1993. 

*13-(6-nitro-1H-indol-1-yl)-matrine* (**3m**). Sophocarpine (73.9 mg, 0.3 mmol) was treated with 6-nitroindole (58.4 mg, 0.36 mmol) according to the general procedure, then purified by silica gel column chromatography with dichloromethane/methanol as the eluents to give the desired product **3m** as a yellow solid, yield: 61.3%; ^1^H-NMR (600 MHz, MeOD-*d*_4_) δ 8.52 (s, 1H, 7’-CH), 7.93 (d, *J* = 8.8 Hz, 1H, 4’-CH), 7.67 (dd, *J* = 8.9, 2.4 Hz, 1H, 5’-CH), 7.60 (t, *J* = 3.1 Hz, 1H, 2’-CH), 6.78 (t, *J* = 3.0 Hz, 1H, 3’-CH), 5.13–5.10 (m, 1H, 13-CH), 4.33 (dd, *J* = 9.4, 6.4 Hz, 1H, 17α-CH_2_), 3.94–3.90 (m, 1H, 11-CH), 3.12 (t, *J* = 12.5 Hz, 1H, 17β-CH_2_), 2.99 (dd, *J* = 17.4, 5.3 Hz, 1H, 14α-CH_2_), 2.86–2.73 (m, 3H, 14β-CH_2_, 10α-CH_2_, 2α-CH_2_), 2.51–2.46 (m, 1H, 12α-CH_2_), 2.25–2.23 (m, 2H, 6-CH, 12β-CH_2_), 1.98–1.90 (m, 4H, 2β-CH_2_, 10β-CH_2_, 5-CH, 7-CH), 1.77–1.31 (m, 8H, 3-CH_2_, 4-CH_2_, 8-CH_2_, 9-CH_2_); ^13^C-NMR (151 MHz, MeOD-*d*_4_) δ 168.77 (15-C), 144.27 (6’-C), 135.68 (8’-C), 134.79 (9’-C), 131.37 (2’-CH), 121.96 (4’-CH), 115.92 (5’-CH), 107.49 (7’-CH), 104.31 (3’-CH), 65.17 (6-CH), 58.15 (2-CH_2_, 10-CH_2_), 52.59 (11-CH), 48.15 (13-CH), 43.57 (17-CH_2_), 42.39 (7-CH), 38.85 (14-CH_2_), 37.09 (5-CH), 31.28 (12-CH_2_), 28.65 (4-CH_2_), 27.44 (8-CH_2_), 22.05 (3-CH_2_), 21.41 (9-CH_2_); ESI-HRMS *m*/*z* Calcd for C_23_H_29_N_4_O [M + H]^+^, 409.2234; found 409.2225. 

*13-(1H-pyrrolo[2,3-c]pyridin-1-yl)-matrine* (**3n**). Sophocarpine (73.9 mg, 0.3 mmol) was treated with 6-azaindole (42.5 mg, 0.36 mmol) according to the general procedure, then purified by silica gel column chromatography with dichloromethane/methanol as the eluents to give the desired product **3n** as a yellow solid, yield: 33.4%; ^1^H-NMR (600 MHz, MeOD-*d*_4_) δ 8.29 (d, *J* = 5.9 Hz, 1H, 7’-CH), 7.98 (d, *J* = 8.9 Hz, 1H, 4’-CH), 7.56 (d, *J* = 3.4 Hz, 1H, 2’-CH), 7.19 (t, *J* = 5.4 Hz, 1H, 5’-CH), 6.64 (d, *J* = 3.2 Hz, 1H, 3’-CH), 5.00 (ddd, *J* = 9.2, 8.6, 4.3 Hz, 1H, 11-CH), 4.31 (dd, *J* = 12.6, 4.5 Hz, 1H, 17α-CH_2_), 3.89 (dt, *J* = 11.3, 5.6 Hz, 1H, 11-CH), 3.11 (t, *J* = 11.8 Hz, 1H, 17β-CH_2_), 2.94 (dd, *J* = 17.3, 5.5 Hz, 1H, 14α-CH_2_), 2.88–2.72 (m, 3H, 14β-CH_2_, 10α-CH_2_, 2α-CH_2_), 2.53–2.49 (m, 1H, 12α-CH_2_), 2.23–2.17 (m, 2H, 6-CH, 12β-CH_2_), 2.03–1.94 (m, 2H, 2β-CH_2_, 10β-CH_2_), 1.89–1.84 (m, 2H, 5-CH, 7-CH), 1.76–1.30 (m, 8H, 3-CH_2_, 4-CH_2_, 8-CH_2_, 9-CH_2_); ^13^C-NMR (151 MHz, MeOD-*d*_4_) δ 168.97 (15-C), 147.24 (5’-CH), 143.47 (7’-CH), 130.56 (8’-C), 129.61 (2’-CH), 119.17 (4’-CH), 117.71 (9’-C), 103.06 (3’-CH), 65.19 (6-CH), 58.18 (2-CH_2_, 10-CH_2_), 52.54 (11-CH), 48.15 (13-CH), 43.57 (17-CH_2_), 42.58 (7-CH), 38.66 (14-CH_2_), 37.16 (5-CH), 31.21 (12-CH_2_), 28.69 (4-CH_2_), 27.47 (8-CH_2_), 22.08 (3-CH_2_), 21.42 (9-CH_2_); ESI-HRMS *m*/*z* Calcd for C_24_H_29_N_4_O [M + H]^+^, 365.2336; found 365.2331. 

### 3.3. BiologyAassay

#### 3.3.1. Cell Culture

Human fetal lung fibroblasts (MRC-5 cells) were purchased from Chinese National Infrastructure of Cell Line Resource and cultured in MEM-NEAA medium containing 10% fetal bovine serum (FBS) in 5% CO_2_ atmosphere at 37 °C. MRC-5 cells in passage between 21 to 26 were used for experiments. 

#### 3.3.2. Sircol Collagen Assay

MRC-5 cells in 1 × 10^5^ cells/mL density were seeded in TC treated 96-well plates and allowed to reach confluence for 48 h. The cells were then starved using MEM-NEAA containing 0.2% BSA for 24 h. After starvation, the medium was changed to MEM-NEAA supplemented with 0.2% BSA, 100 μM L-Proline, 100 μM Ascorbic acid, with or without 10 ng/mL TGF-β1 (Peprotech) in the presence of vehicle or different concentrations of compounds. After 48 h incubation, the MRC-5 cells were washed with phosphate buffered saline (PBS) and fixed in −20 °C precooled methanol for 10 min. The cells were than washed with PBS before incubation in 0.1% PSR staining solution (0.1% Sirus red in saturated water solution of picric acid) for 1 h. The staining solution was then removed and the cells were washed with 0.1% acetic acid for three times. 200 μL of 0.1 mol/L sodium hydroxide solution were added to each well and the plates were placed on a rocking platform for 1 h. The optical density (OD) values at 550 nm were determined with an Infinite 2000 spectrophotometer. 

#### 3.3.3. Cytotoxicity Assay

MRC-5 cells were incubated with different concentrations of compounds for 48 h, and then 10 μL of CCK-8 solution were added to each well. The cells were incubated at 37 °C for another 3 h before determining the OD values at 492 nm. DMSO at 0.1% *v*/*v* final concentration was used to solubilize the test compounds, except for compound **3b**, **3c**, and **3e**, where 1% *v*/*v* DMSO was used to ensure complete solubilization. This concentration was based on our preliminary findings and reference [29] which suggested that exposure of human dermal fibroblast cultures for 48 h to concentrations reaching 1% *v*/*v* DMSO had no significant effect on cell viability. 

#### 3.3.4. Immunofluorescence Assay

MRC-5 cells were seeded on 0.2% gelatin-coated glass coverslips and treated with or without 10 ng/mL TGF-β1 in the presence of vehicle or 10 μM **3f** for 48 h. The cells were washed with PBS and fixed in freshly prepared 4% paraformaldehyde (PFA) at room temperature for 10 min. The cells were then permeabilized with 0.25% Triton X-100 for 10 min before 30 min of blocking by PBS solution supplemented with 1% BSA and 22.5 mg/mL glycine (the permeabilization was omitted for S100A4 and TGFβRI staining). The cells were incubated with primary antibody overnight at 4 °C and labeled with AlexaFluor 488-conjugated or Dylight 488-conjugated secondary antibody. The antibodies used in this study were listed as follows: anti-COL1A1 (1:100 dilution; Boster Biological Technology, Wuhan, China; #BA0326); anti-COL3A1 (1:100 dilution; Boster Biological Technology, Wuhan, China; #BM1625); anti-Fibronectin (1:400 dilution; Boster Biological Technology, Wuhan, China; #BA1771); anti-αsma (1:100 dilution; Boster Biological Technology, Wuhan, China; #BM0002); anti-S100A4 (1:50 dilution; Boster Biological Technology, Wuhan, China; # BM4636); anti-TGFβRI (1:100 dilution; Boster Biological Technology, Wuhan, China; #BA0294); anti-Smad2/3 (1:100 dilution; Boster Biological Technology, Wuhan, China; #BA1395); AlexaFluor 488-conjugated goat anti-rabbit IgG preabsorbed antibody (1:200 dilution; Abcam, Cambridge, UK; #ab15065); Dylight 488-conjugated goat anti-mouse IgG antibody (1:200 dilution; Boster Biological Technology, Wuhan, China; #BA1126). Nuclei were stained with Prolong diamond antifade mountant with DAPI (Invitrogen, Carlsbad, CA, USA; P36966). The photos were taken on a Zeiss observer D1 epifluorescence microscope. The mean fluorescence intensity (MFI) of each photo was calculated by ImageJ. Student’s *t*-test was used to evaluate difference between groups. Differences were considered as statistically significant at *p*-values less than 0.05. 

#### 3.3.5. Scratch Assay

MRC-5 cells were seeded on 6-well plates and allowed to reach confluence for 48 h. The cell monolayer was gently scratched with a 1 mL pipette tip across the center of the well. After scratching, the cells were washed twice with medium to remove detached cells. The cells were then treated with or without 10 ng/mL TGF-β1 in the presence of vehicle or 10 μM **3f** for 24 h. The gap area in wound region was quantitatively evaluated using ImageJ [30] and MRI wound healing tool plugin [31]. 

## 4. Conclusions

In summary, a total of 18 matrine derivatives were designed, synthesized, and evaluated for their anti-fibrotic activities. Among the new derivatives, compound **3f** exhibited the most potent inhibitory effect against TGF-β1-induced total collagen accumulation with the IC_50_ value of 3.3 μM, which was 266-fold more potent than matrine or 400-fold more potent than Pirfenidone. Anti-fibrotic mechanism study suggested that **3f** could significantly inhibit the fibroblast-to-myofibroblast transition and extracellular matrix production in MRC-5 cells. The TGF-β/Smad signaling was also repressed by **3f**, as evidenced by inhibition of cytoplasm-to-nuclear translocation of Smad2/3 and suppression of TGF-β1-induced upregulation of TGFβRI. Additionally, **3f** exhibited potent inhibitory effect against TGF-β1-induced fibroblasts migration. Overall, this work provided useful information for further structural modification and development of matrine-based anti-pulmonary fibrosis agents.

## Figures and Tables

**Table 1:**
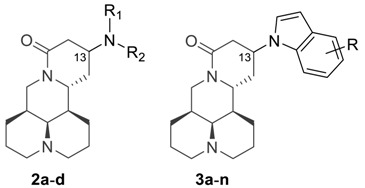
Anti-fibrotic activity, cytotoxicity, and selectivity index of matrine derivatives.

Code	R	IC_50_ (μM) ^a^	CC_50_ (μM) ^b^	SI ^c^	13*S*/13*R* ratio ^d^	ClogP ^e^
**2a**	R_1_ = Me, R_2_ = H	65.3 ± 4.2	609.3 ± 29.8	9.3	pure 13*S*	0.75
**2b**	R_1_ = R_2_ = Me	92.1 ± 1.9	624.5 ± 13.2	6.8	pure 13*S*	1.33
**2c**	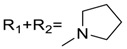	255.8 ± 22.1	675.8 ± 49.1	2.6	2.6:1	1.89
**2d**	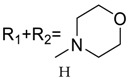	138.4 ± 9.8	507.2 ± 25.3	3.7	2.4:1	0.92
**3a**	H	28.3 ± 3.9	106.6 ± 5.9	3.8	8.1:1	3.28
**3b**	3′-Me	39.0 ± 0.6	95.1 ± 6.5	2.4	5.7:1	3.78
**3c**	4′-OMe	44.7 ± 3.6	56.7 ± 3.9	1.3	4.9:1	3.34
**3d**	4′-NO_2_	4.3 ± 0.4	32.8 ± 2.7	7.6	10.1:1	3.11
**3e**	5′-OMe	68.1 ± 5.5	127.5 ± 1.4	1.9	7.3:1	3.34
**3f**	5′-Cl	3.3 ± 0.3	26.7 ± 0.7	8.0	pure 13*S*	4.03
**3g**	5′-Br	6.5 ± 1.2	18.2 ± 0.6	2.8	4.9:1	4.18
**3h**	5′-CN	72.1 ± 4.0	153.6 ± 11.0	2.1	1.6:1	2.81
**3i**	5′-NO_2_	14.5 ± 1.0	65.4 ± 3.1	4.5	3.3:1	3.11
**3j**	5′-aza	16.7 ± 3.5	114.0 ± 5.9	6.8	pure 13*S*	1.98
**3k**	6′-F	23.1 ± 5.3	78.1 ± 3.4	3.4	1.9:1	3.46
**3l**	6′-Cl	27.0 ± 1.4	45.8 ± 1.1	1.7	4.9:1	4.03
**3m**	6′-NO_2_	19.3 ± 2.2	53.3 ± 1.9	2.8	11.5:1	3.11
**3n**	6′-aza	69.5 ± 8.2	84.2 ± 2.2	1.2	1.5:1	1.98
Matrine	-	878 ± 68	2466 ± 103	2.8	-	1.36
Sophocarpine	-	1186 ± 135	3160 ± 355	2.7	-	1.36
Pirfenidone	-	1320 ± 98	4159 ± 239	3.1	-	2.40

^a^. Inhibition of TGF-β1 induced total collagen accumulation in MRC-5 cells; ^b^. 50% cytotoxic concentration; ^c^. Selectivity index (SI = CC_50_/IC_50_); ^d^. the ratio of 13*S* isomer vs. 13*R* isomer, determined by ^1^H-NMR spectra; ^e^. ClogP (Calculated log partition coefficient) calculated using ChemDraw Professional.

## Supplementary Materials

The following are available online, ^1^H, ^13^C-NMR spectra of the synthesized matrine derivatives and NOE spectrum of compound **3j**.
