# Supplementary material for: Design and Synthesis of Matrine Derivatives as Novel Anti-Pulmonary Fibrotic Agents via Repression of the TGFβ/Smad Pathway
Source: Molecules. 2019 Mar 20;24(6):1108. doi: 10.3390/molecules24061108 (PMC6470603; doi:10.3390/molecules24061108)

Supplementary information

Design and Synthesis of Matrine Derivatives as Novel Anti-Pulmonary Fibrotic Agents via Repression of the TGFβ/Smad Pathway

Lingyu Li[1], Liyan Ma[1], Dongchun Wang[1], Hongmei Jia[1], Meng Yu[1], Yucheng Gu[2], Hai Shang[1, 3]*, Zhongmei Zou[1]*

[1] Institute of Medicinal Plant Development, Chinese Academy of Medical Sciences and Peking Union Medical College, Beijing 100193, P. R.
[2] Syngenta, Jealott's Hill International Research Centre, Berkshire, UK
[3] State Key Laboratory of Bioactive Substance and Function of Natural Medicines, Institute of Materia Medica, Chinese Academy of Medical Sciences and Peking Union Medical College, Beijing 100050, P. R. China
* Correspondence: zmzou@implad.ac.cn; Tel.: +86-10-5783-3290

Contents:

Page 2-20: NMR spectrum of synthesized compounds

S1-1 ^{1}H NMR spectrum of 2a (MeOD-d_4, 600 MHz)

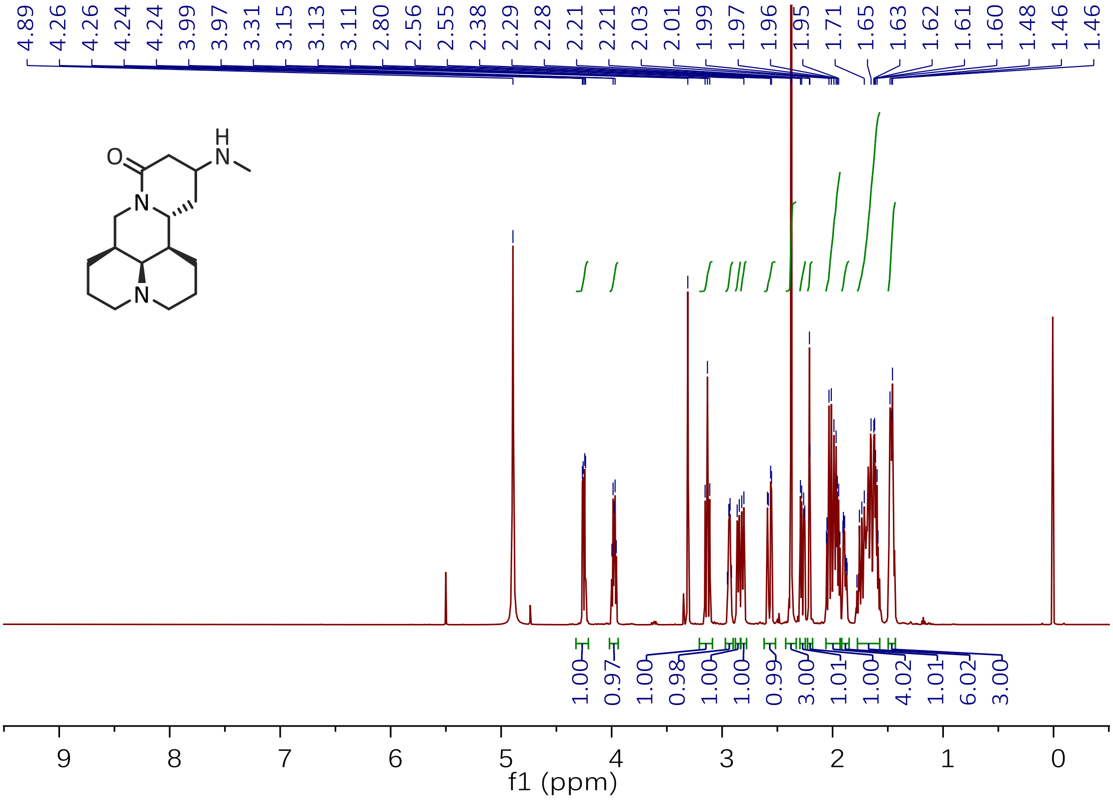

S1-2 ^{13}C NMR spectrum of 2a (MeOD-d_4, 151 MHz)

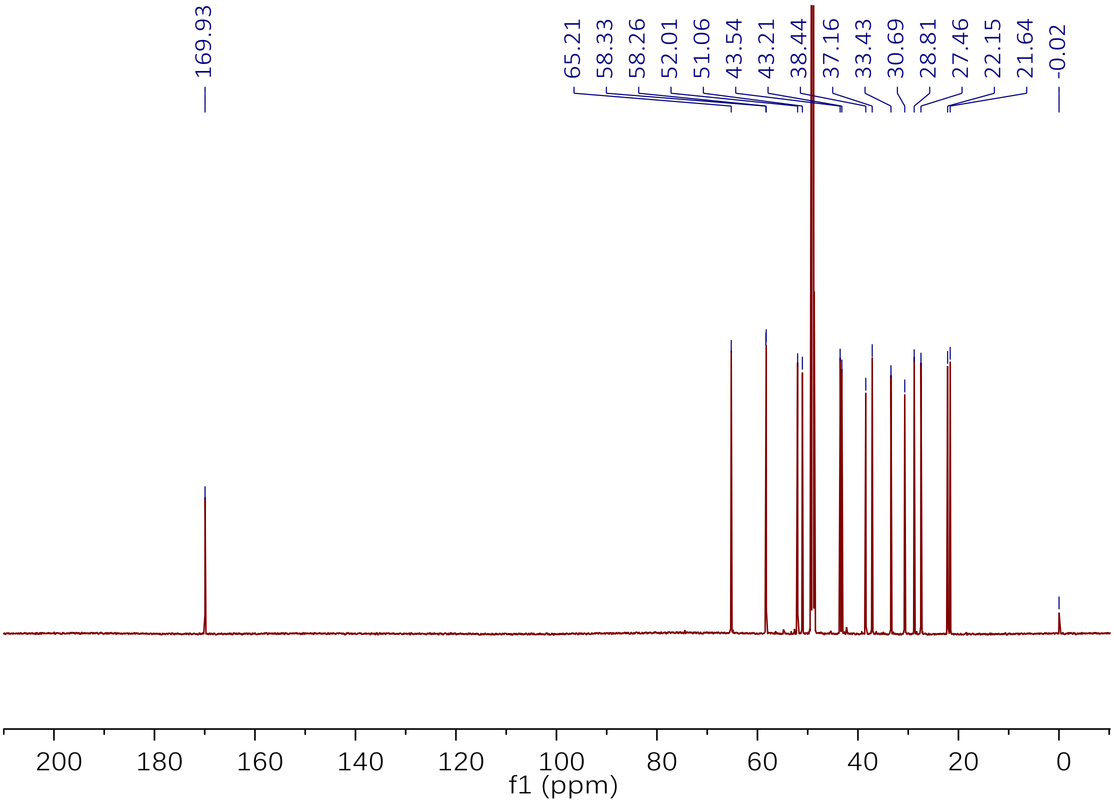

S2-1 ^{1}H NMR spectrum of 2b (MeOD-*d*$_4$, 600 MHz)

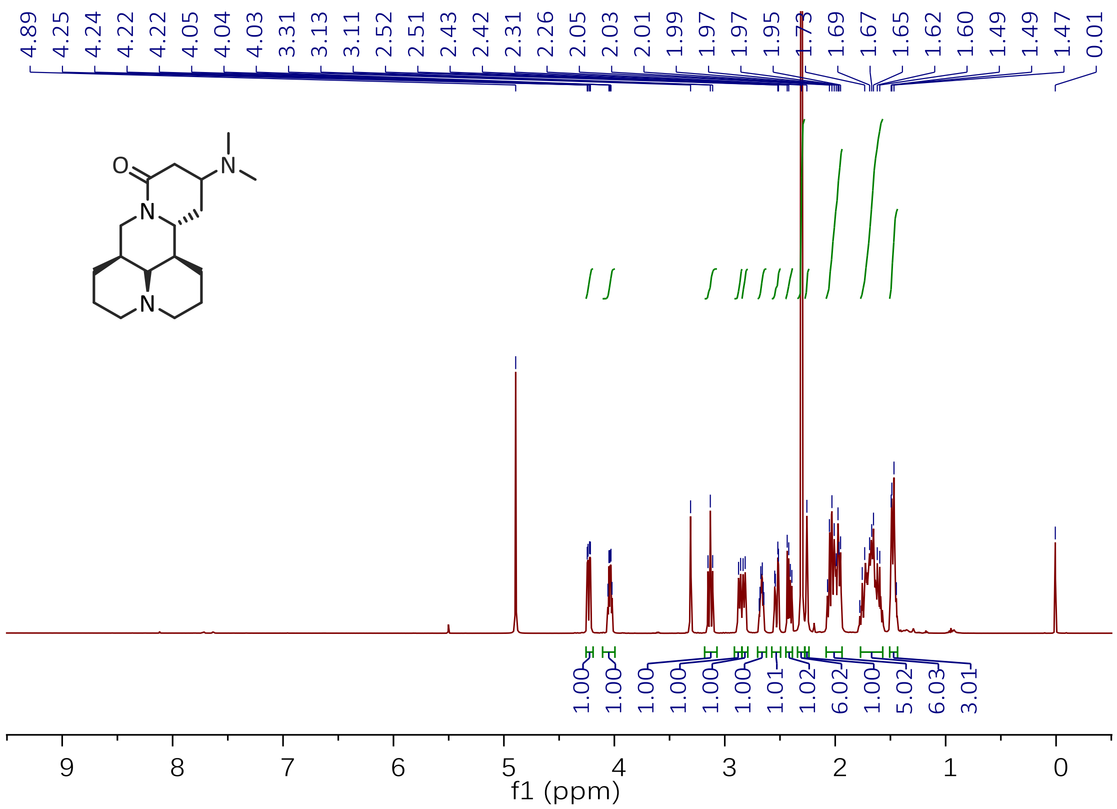

S2-2 ^{13}C NMR spectrum of 2b (MeOD-*d*$_4$, 151 MHz)

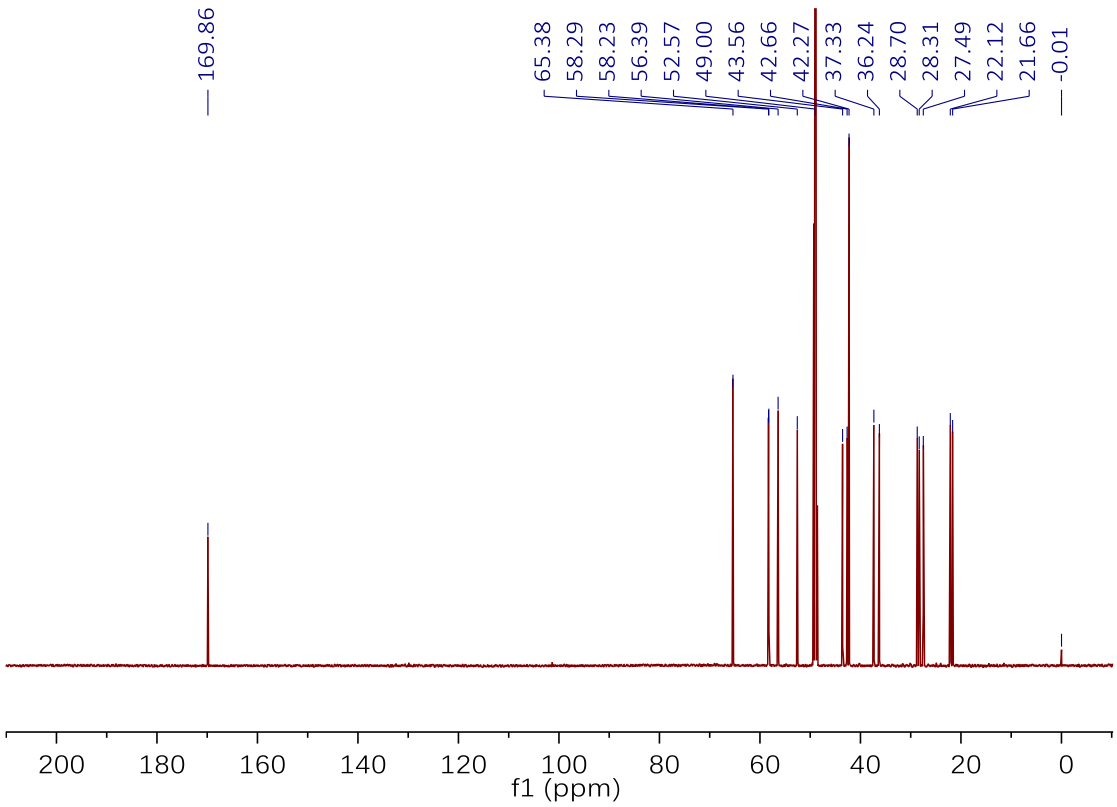

S3-1 ^{1}H NMR spectrum of 2c (MeOD-d_4, 600 MHz)

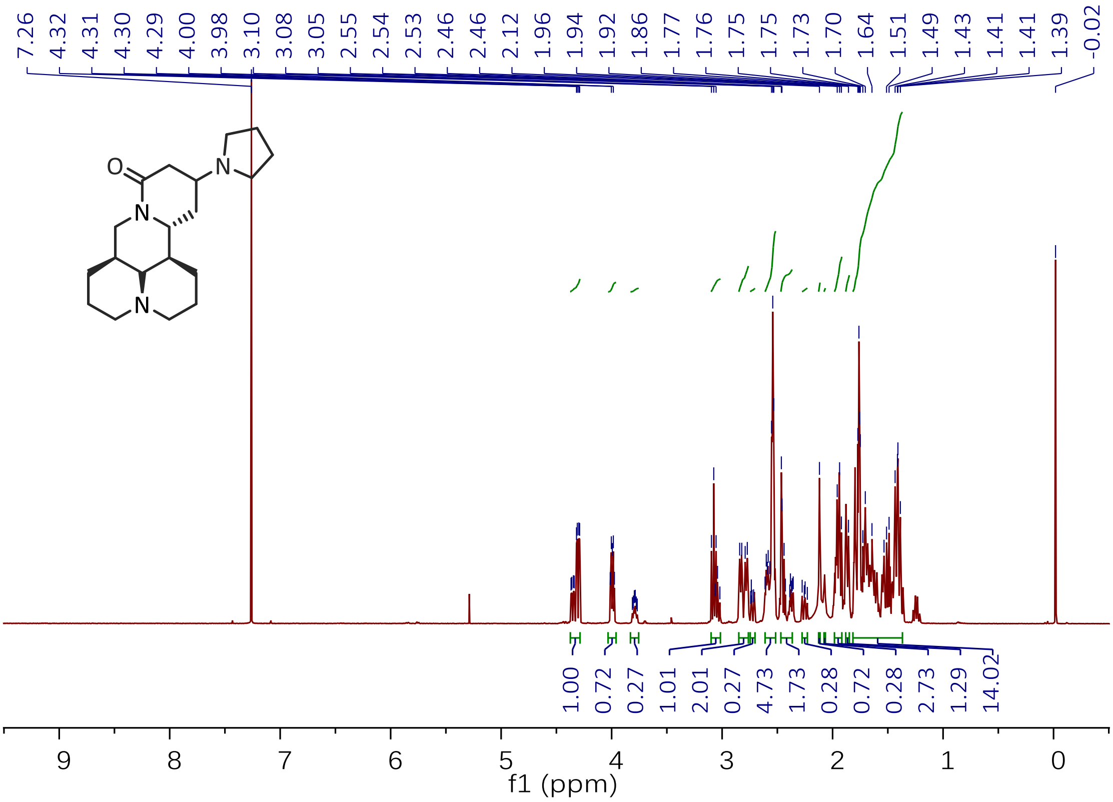

S3-2 ^{13}C NMR spectrum of 2c (MeOD-d_4, 151 MHz)

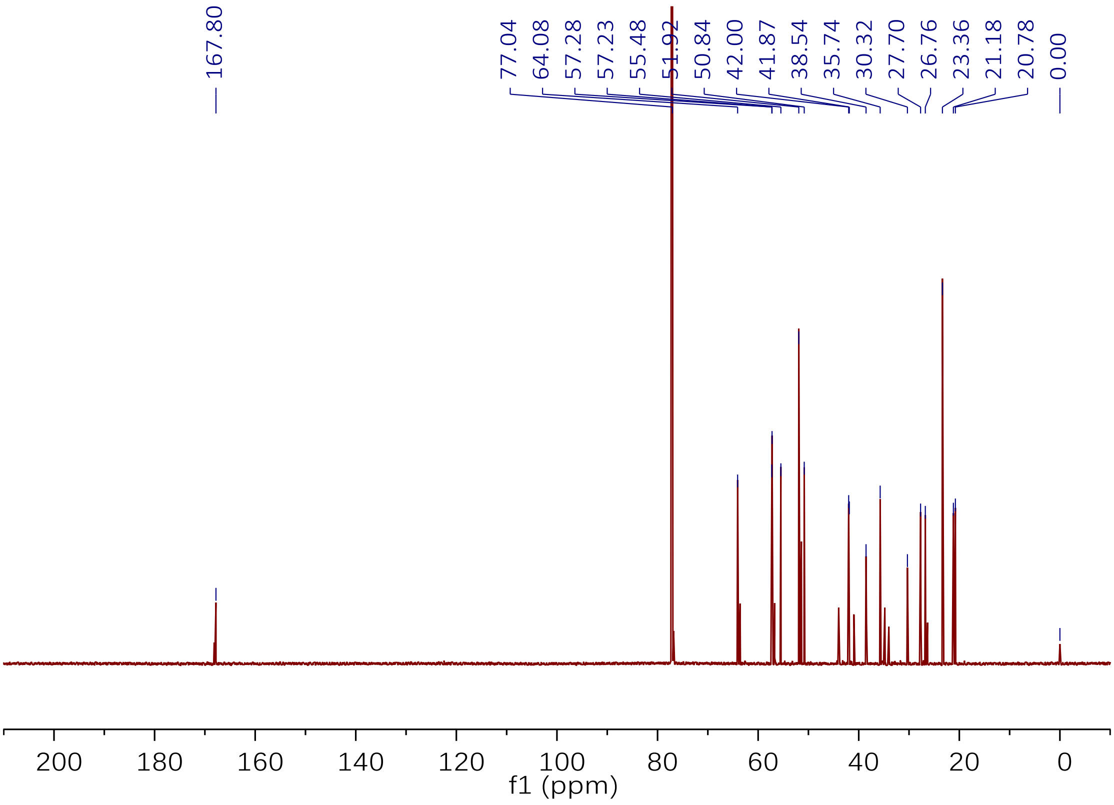

S4-1 ^{1}H NMR spectrum of 2d (MeOD-*d*4, 600 MHz)

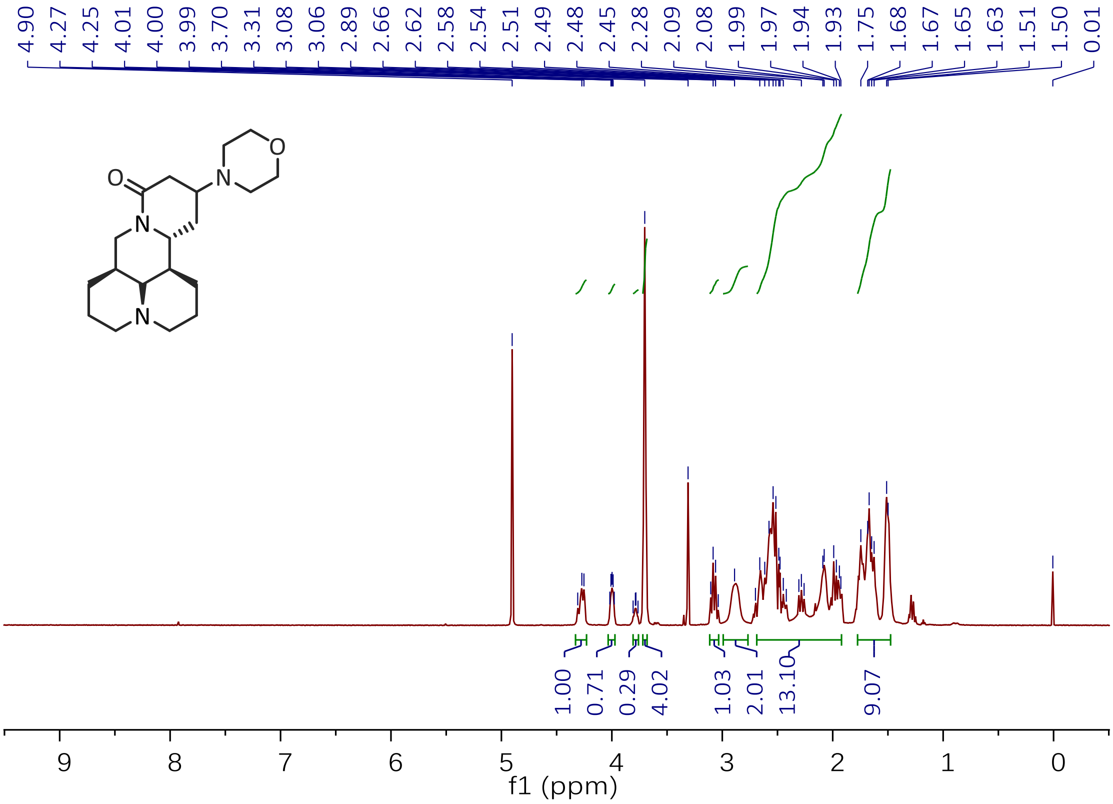

S4-2 ^{13}C NMR spectrum of 2d (MeOD-*d*4, 151 MHz)

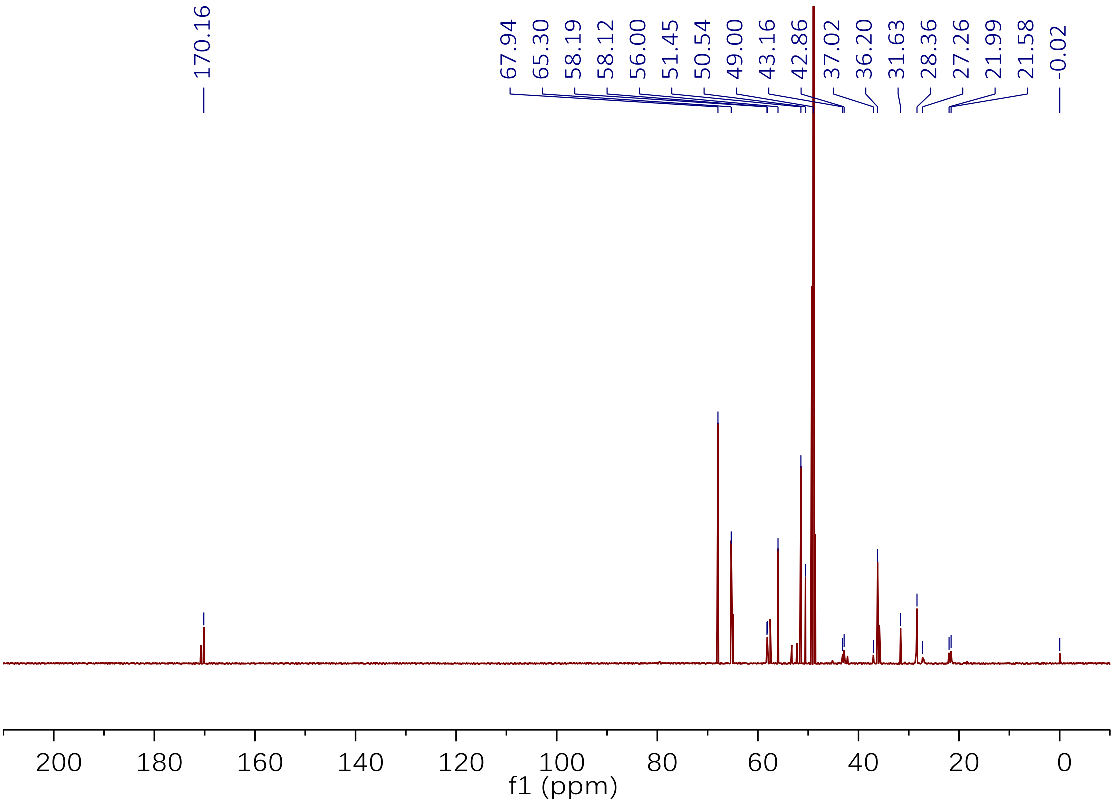

S5-1 ^{1}H NMR spectrum of 3a (MeOD-d_4, 600 MHz)

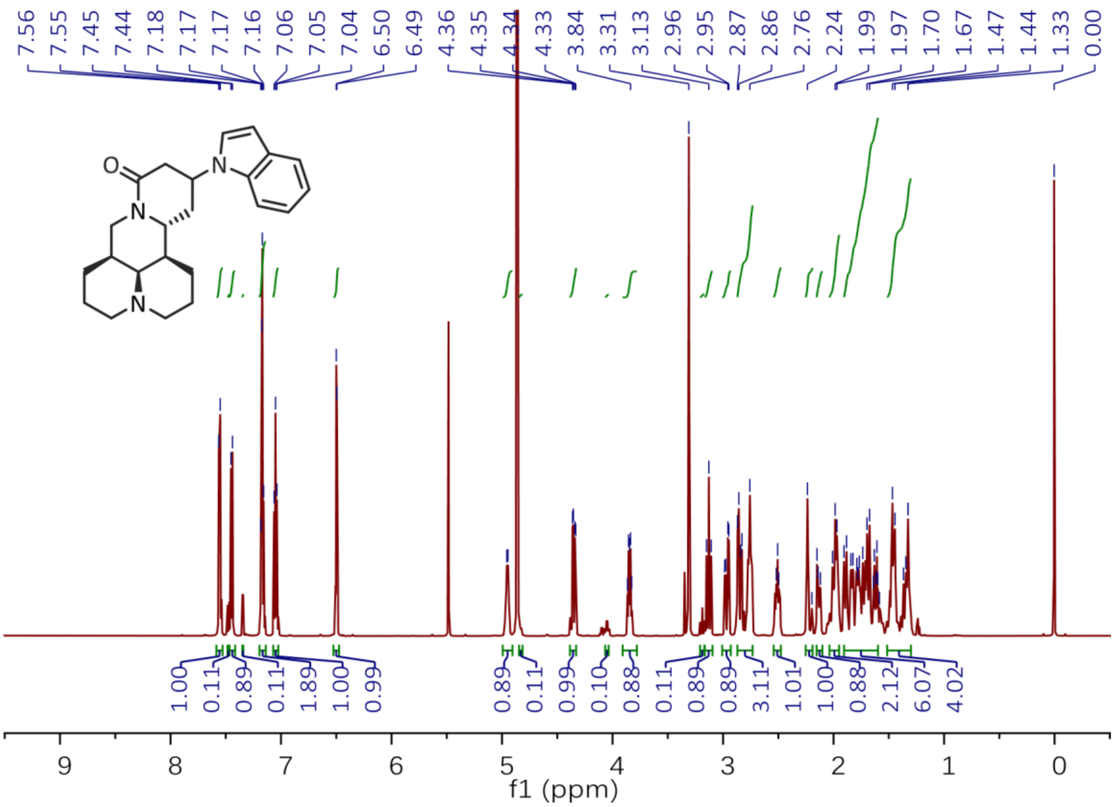

S5-2 ^{13}C NMR spectrum of 3a (MeOD-d_4, 151 MHz)

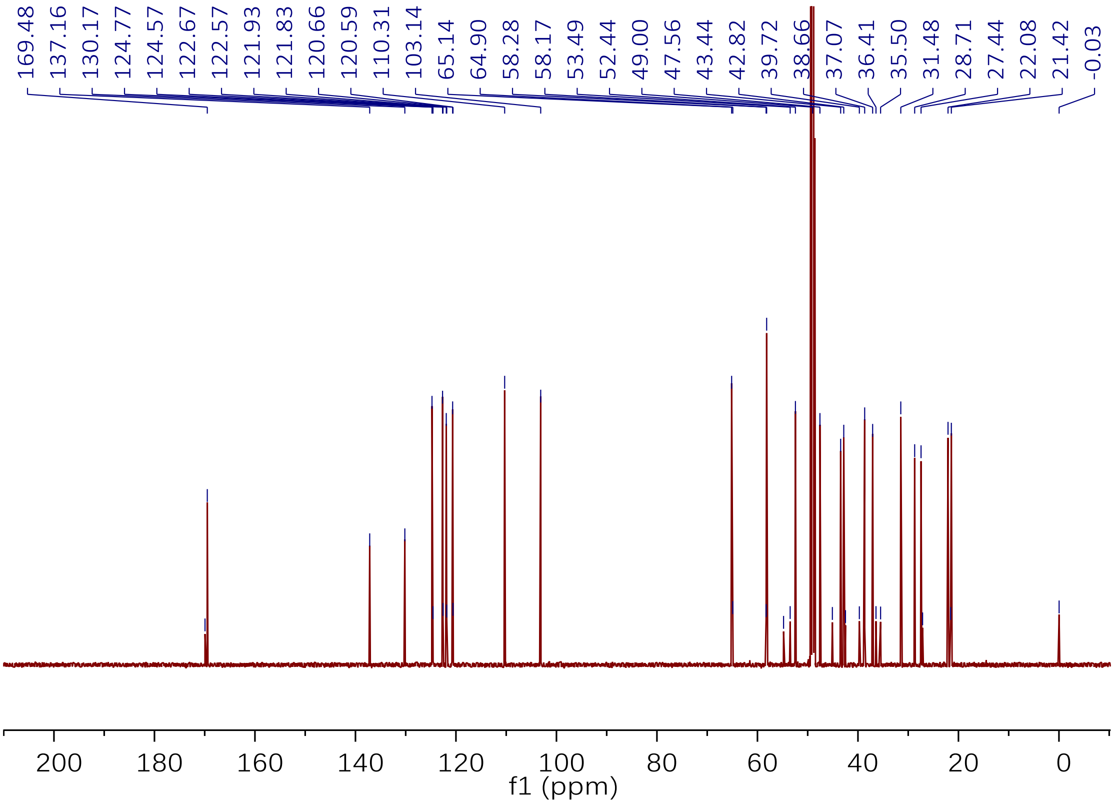

S6-1 ^{1}H NMR spectrum of 3b (MeOD-d_4, 600 MHz)

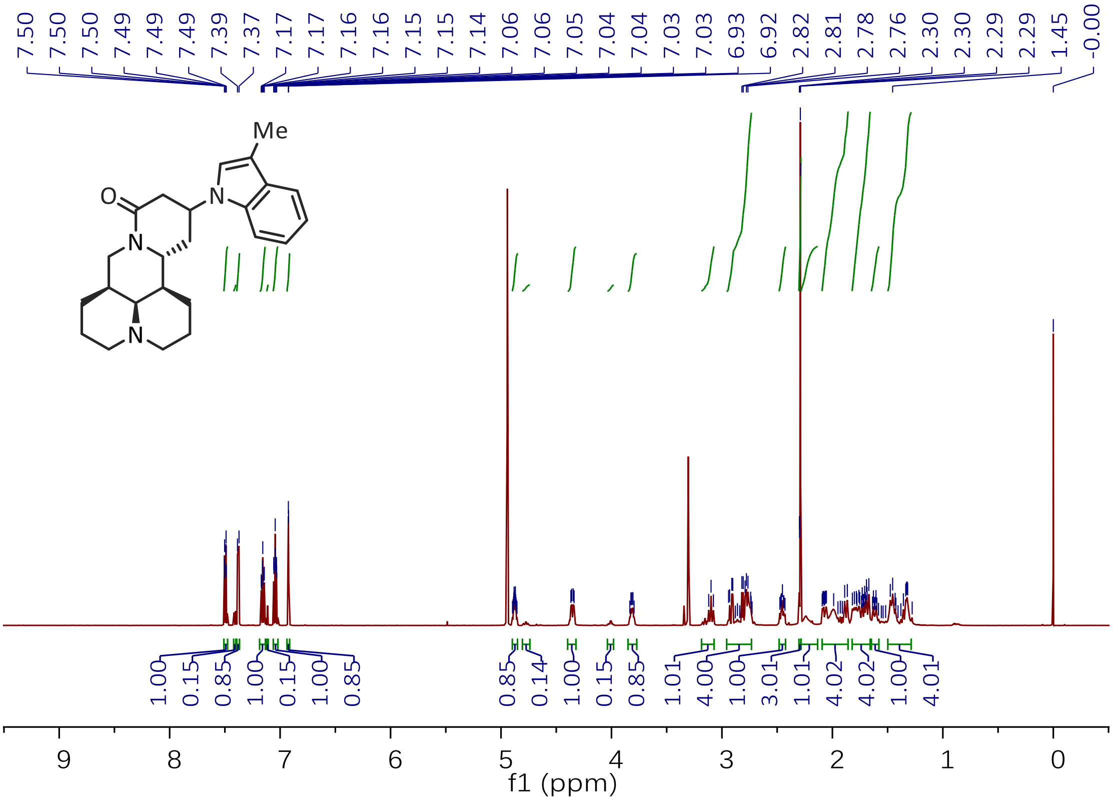

S6-2 ^{13}C NMR spectrum of 3b (MeOD-d_4, 151 MHz)

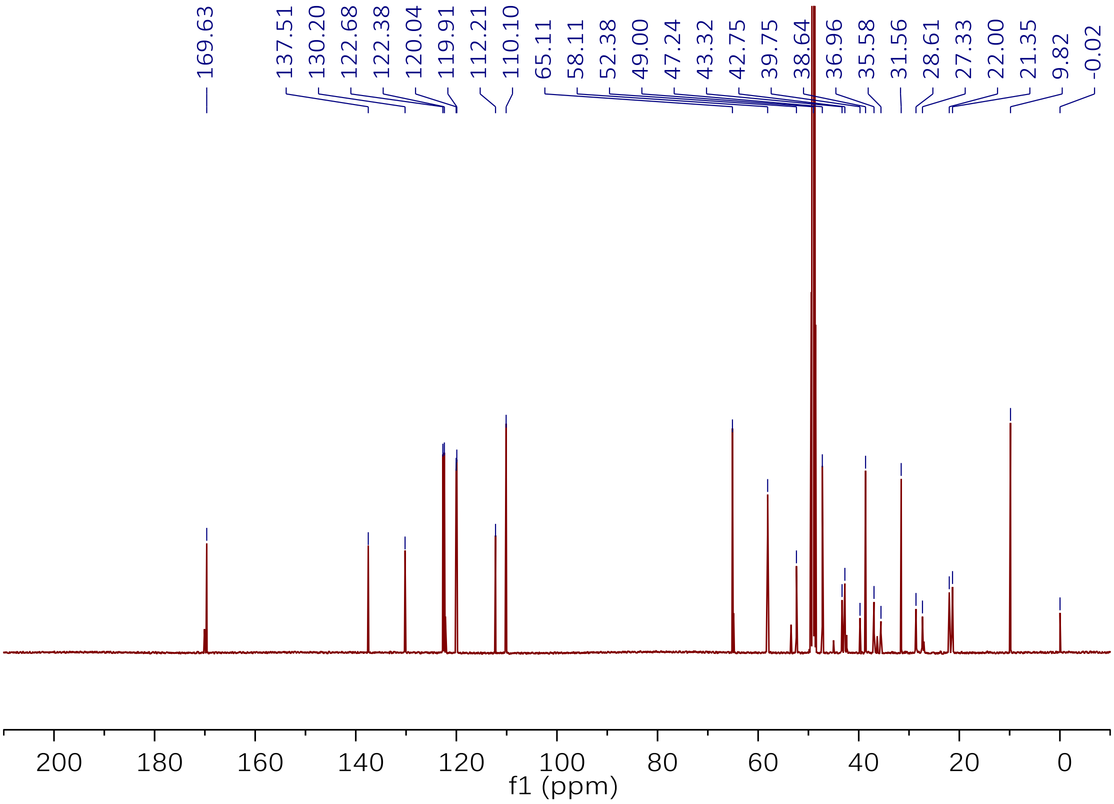

S7-1 ^{1}H NMR spectrum of 3c (MeOD-*d*$_4$, 600 MHz)

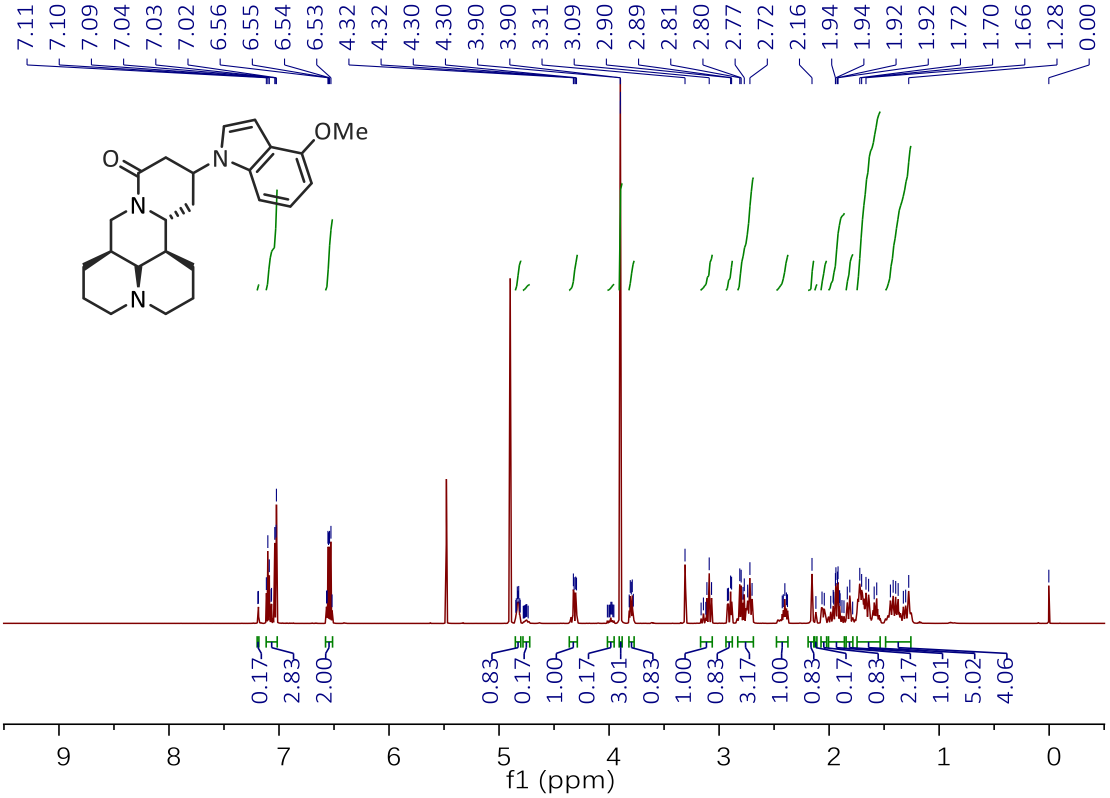

S7-2 ^{13}C NMR spectrum of 3c (MeOD-*d*$_4$, 151 MHz)

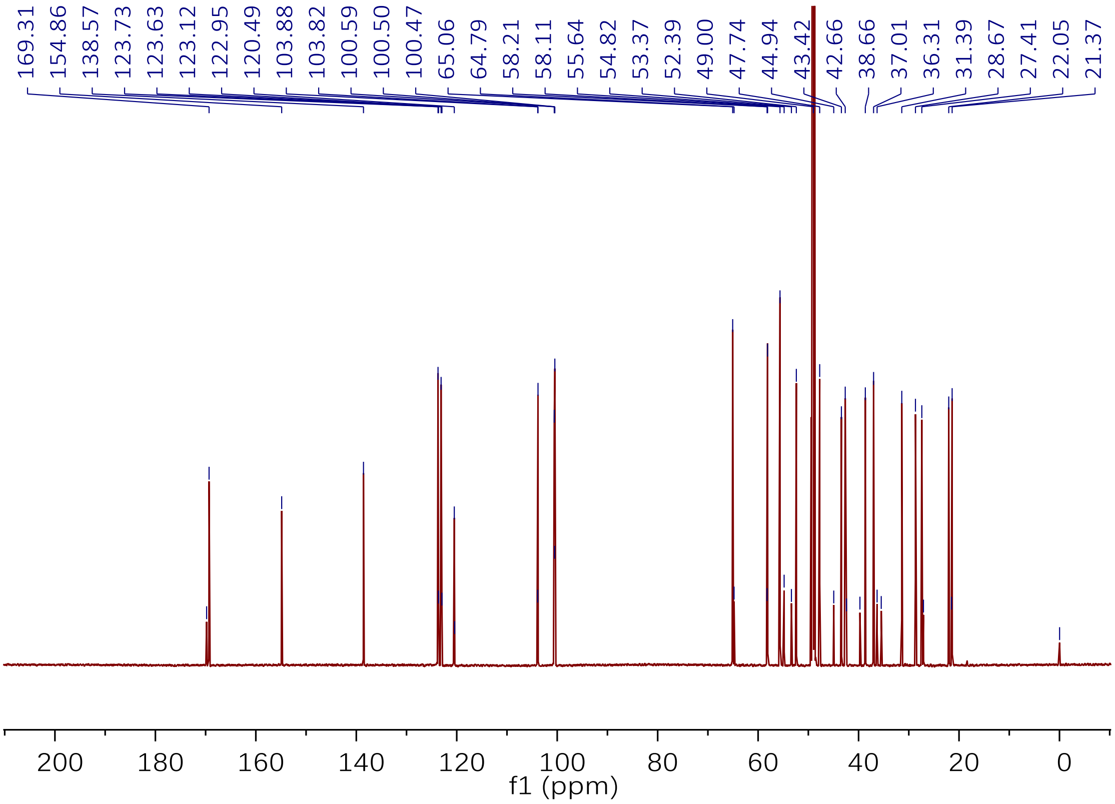

S8-1 ^{1}H NMR spectrum of 3d (MeOD-d_4, 600 MHz)

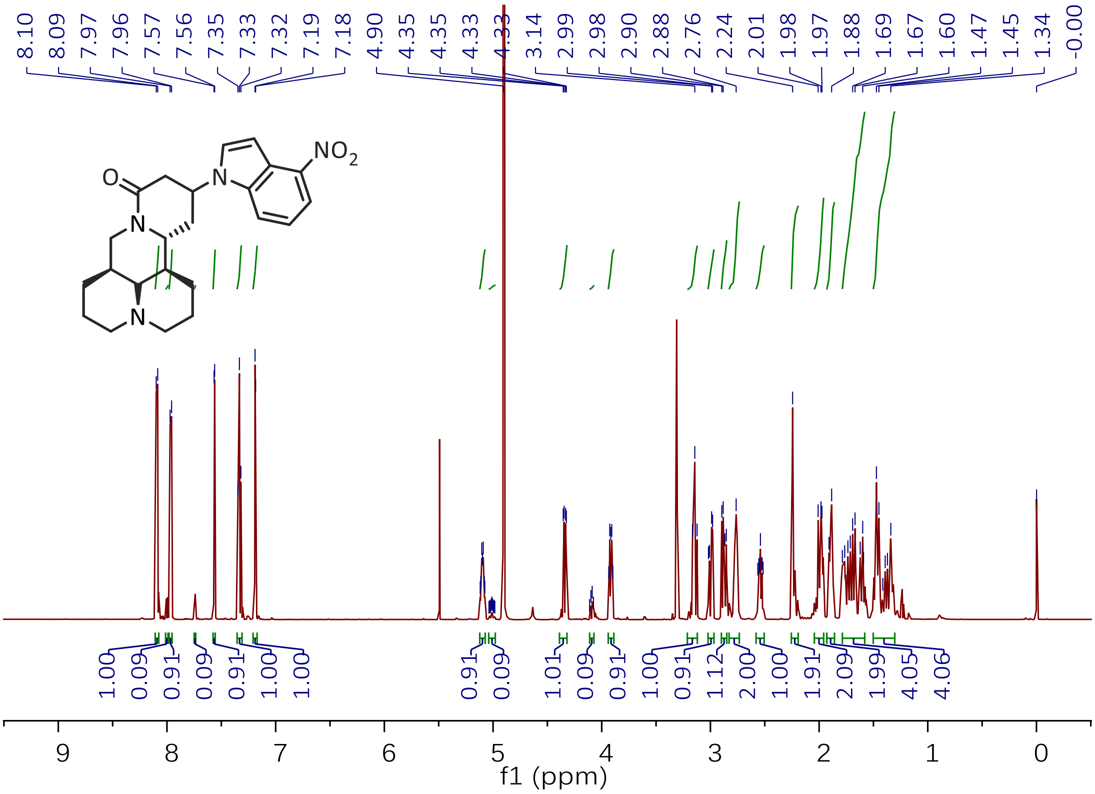

S8-2 ^{13}C NMR spectrum of 3d (MeOD-d_4, 151 MHz)

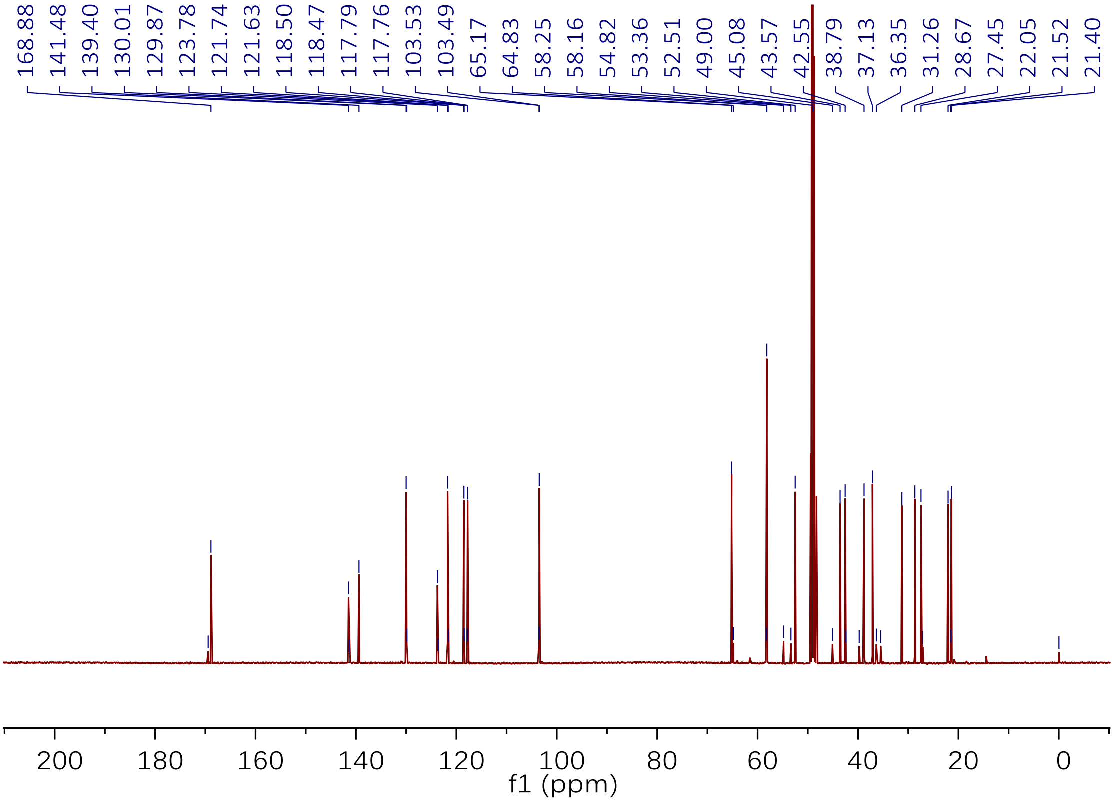

S9-1 ^{1}**H NMR spectrum of 3e (MeOD-*d*$_4$, 600 MHz)**

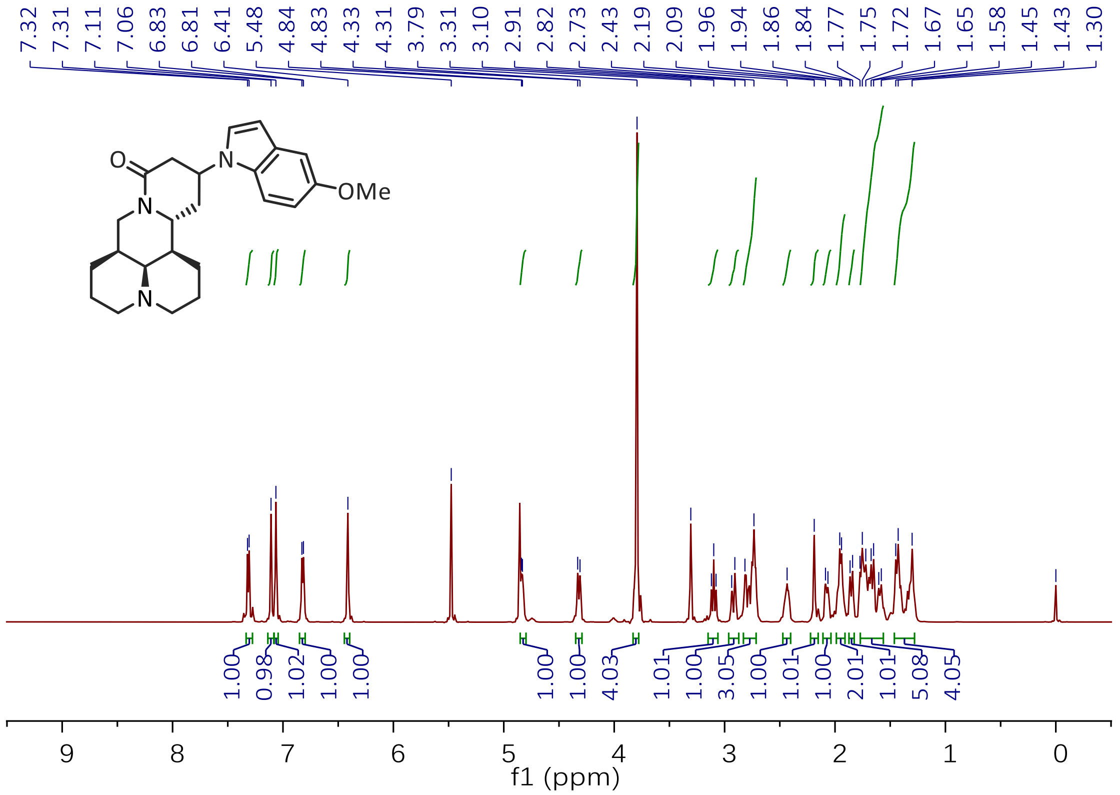

S9-2 ^{13}**C NMR spectrum of 3e (MeOD-*d*$_4$, 151 MHz)**

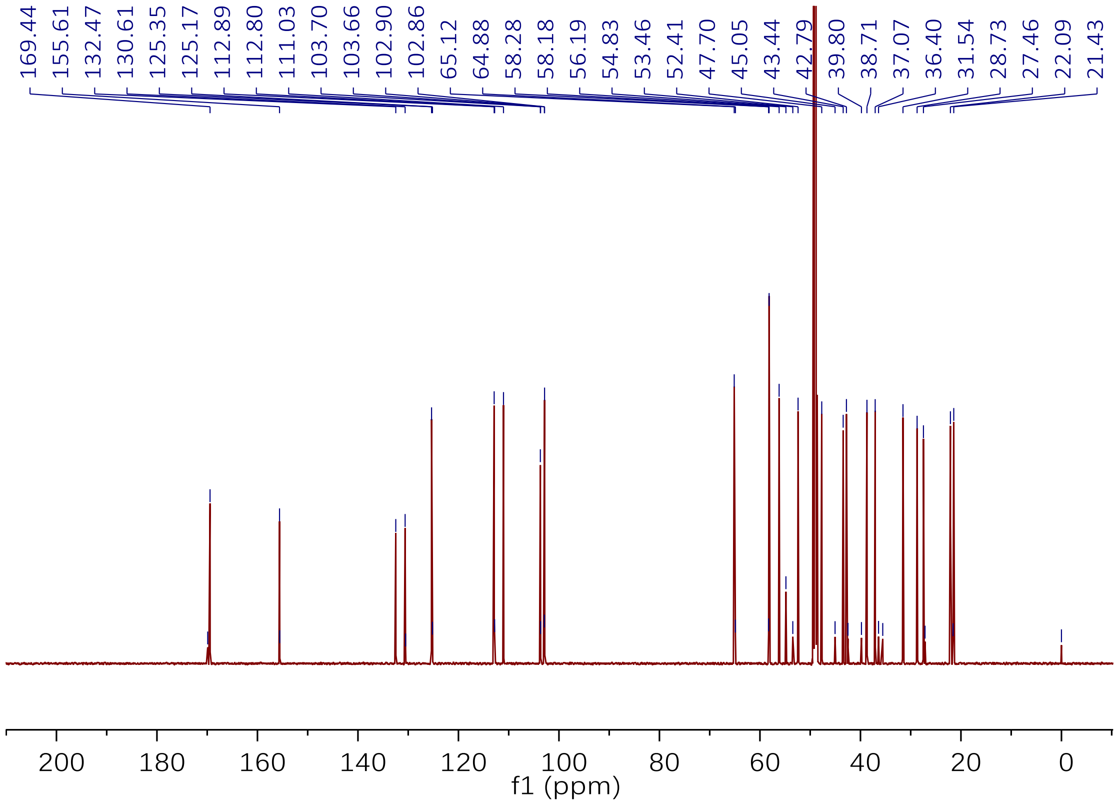

S10-1 ^{1}H NMR spectrum of 3f (MeOD-*d*4, 600 MHz)

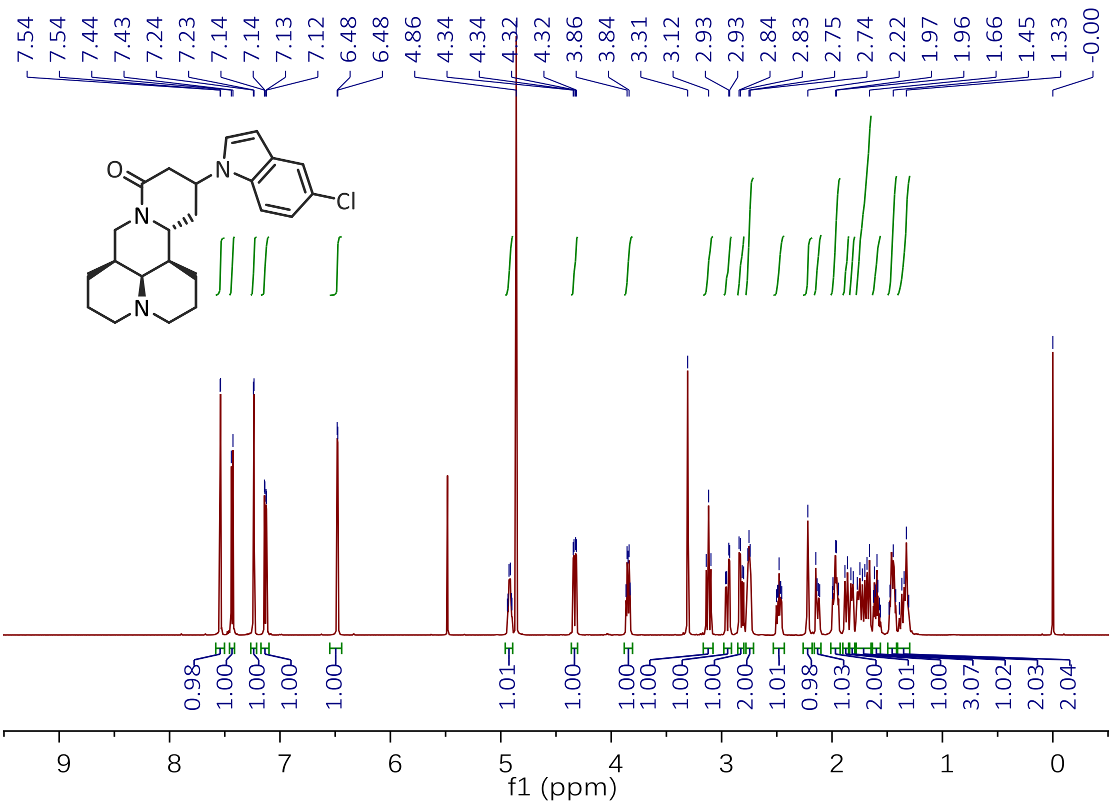

S10-2 ^{13}C NMR spectrum of 3f (MeOD-*d*4, 151 MHz)

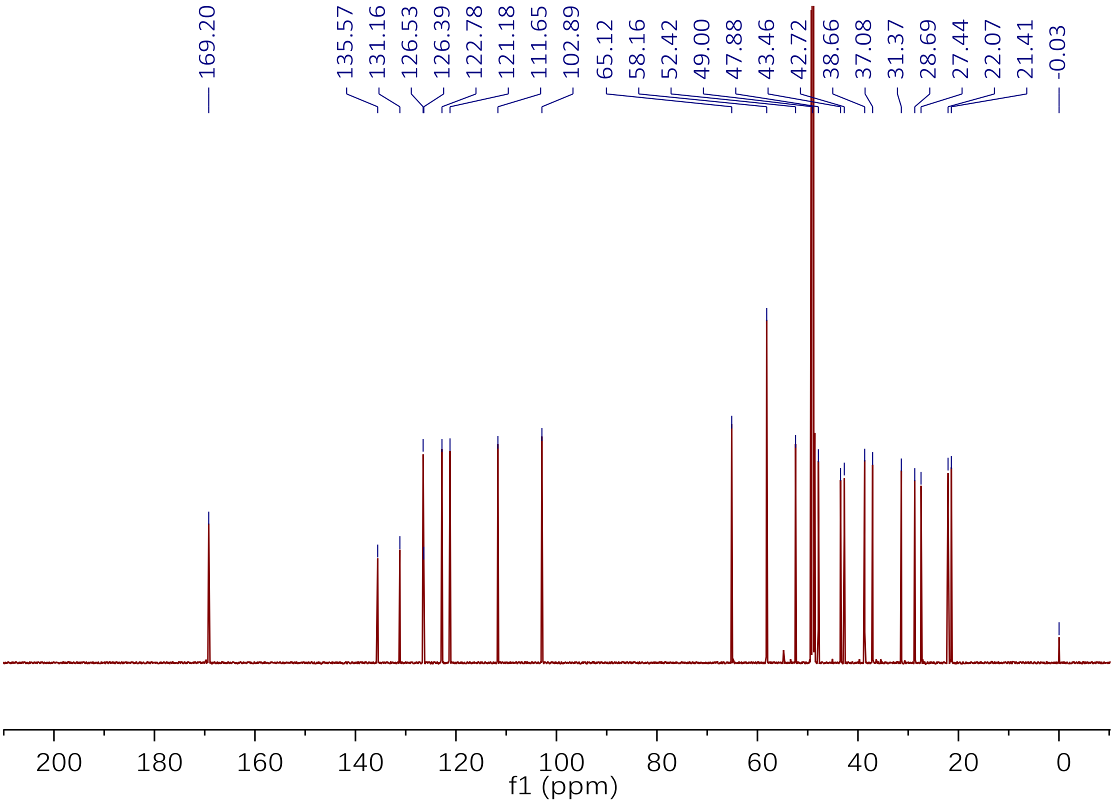

S11-1 ^{1}H NMR spectrum of 3g (MeOD-d_4, 600 MHz)

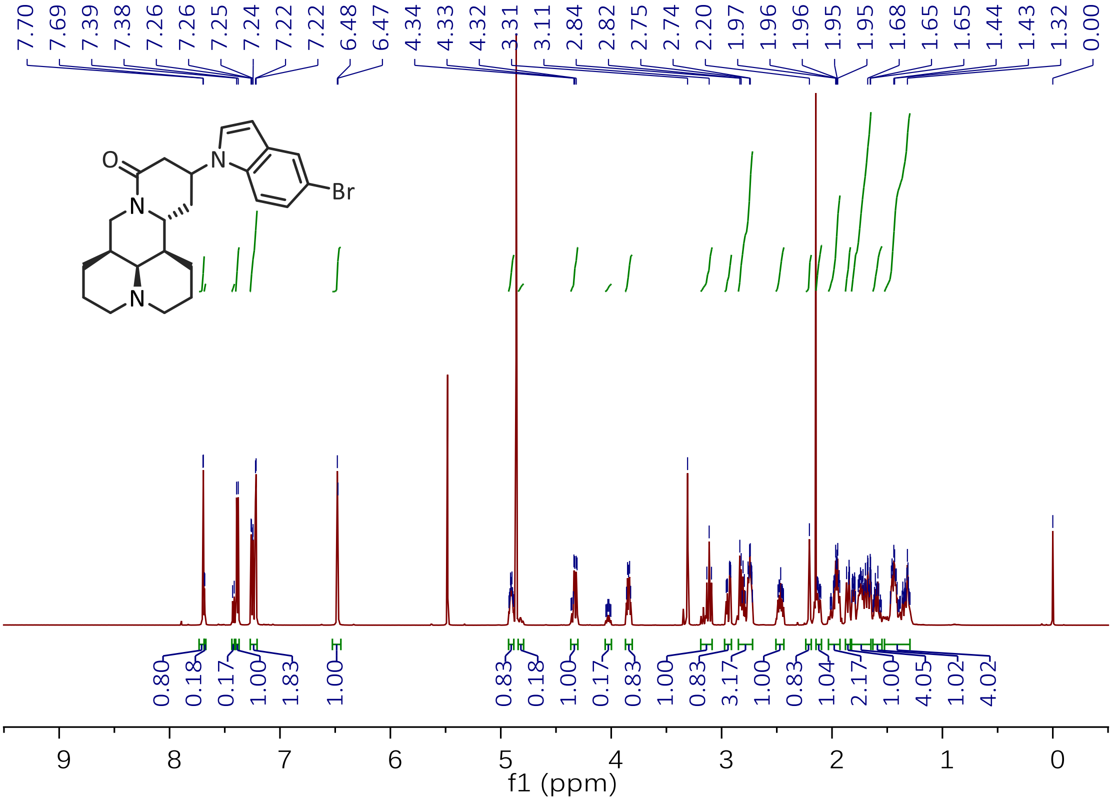

S11-2 ^{13}C NMR spectrum of 3g (MeOD-d_4, 151 MHz)

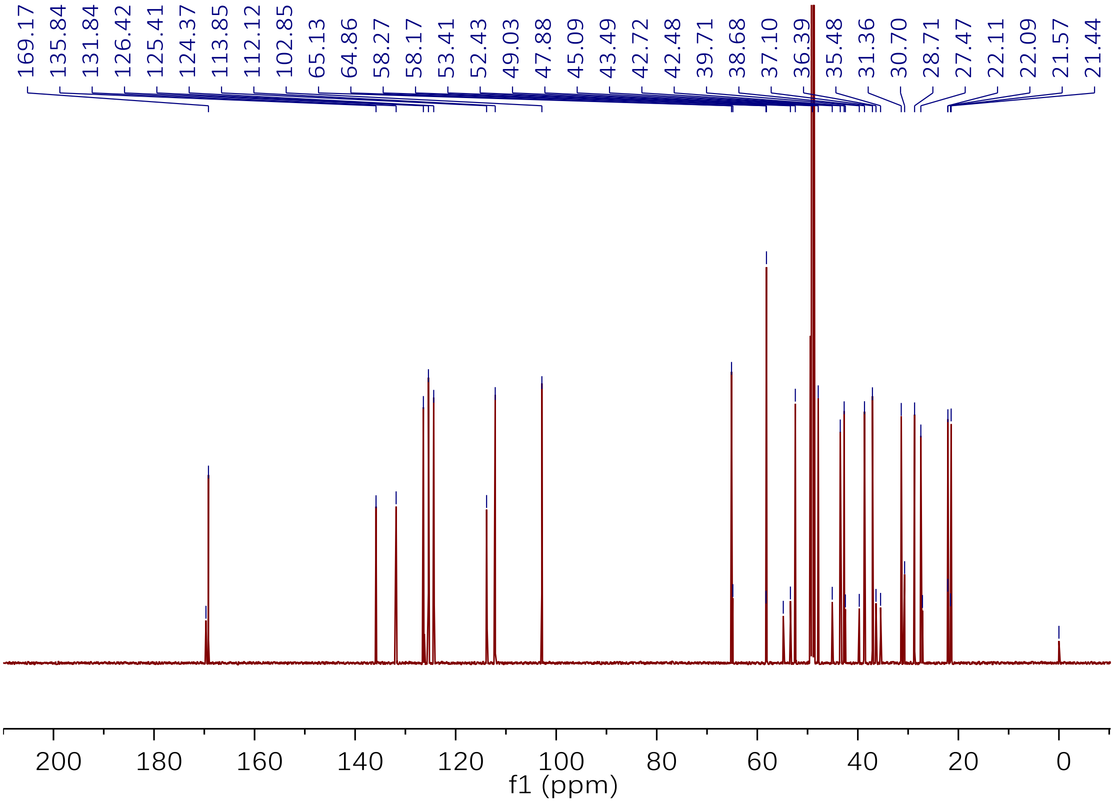

S12-1 ¹H NMR spectrum of 3h (MeOD-*d*₄, 600 MHz)

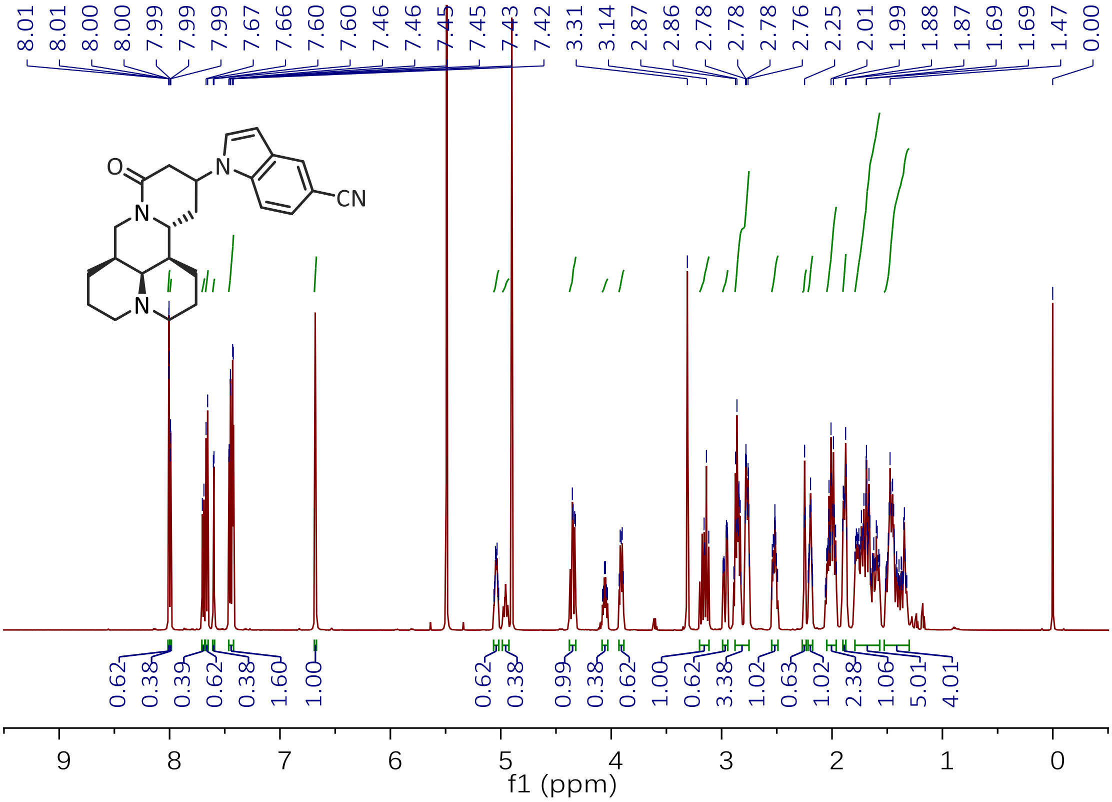

S12-2 ¹³C NMR spectrum of 3h (MeOD-*d*₄, 151 MHz)

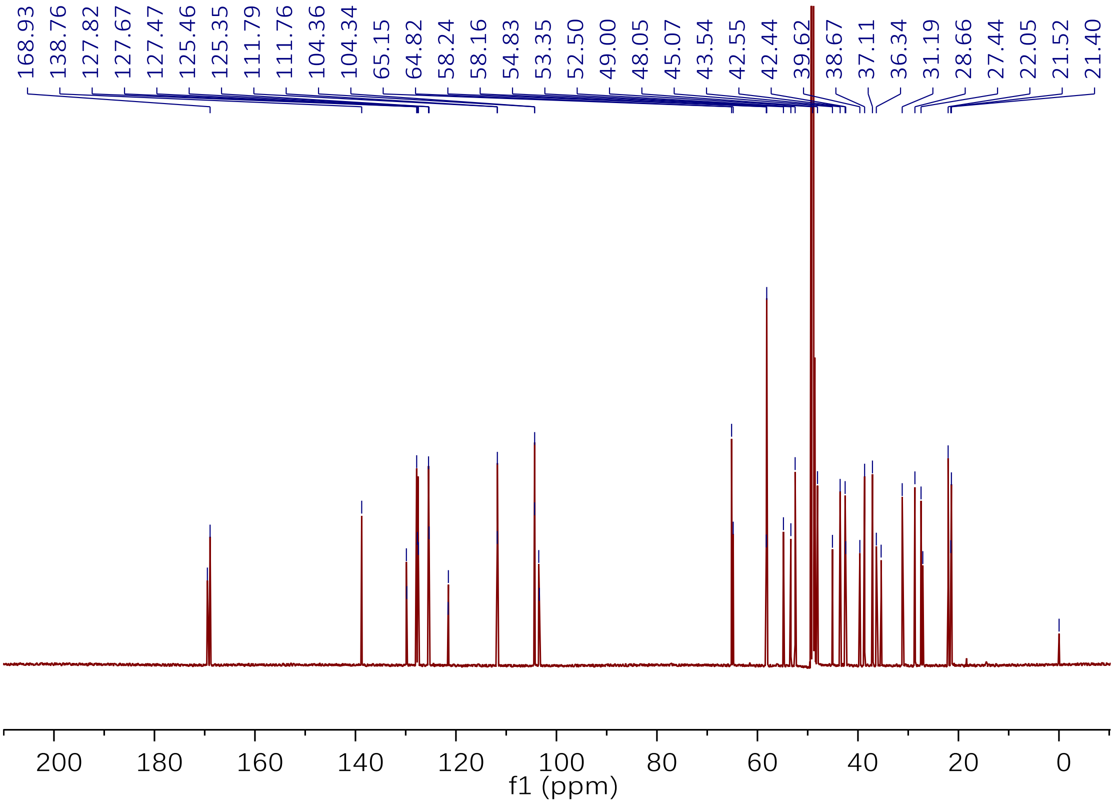

S13-1 ¹H NMR spectrum of 3i (MeOD-*d*₄, 600 MHz)

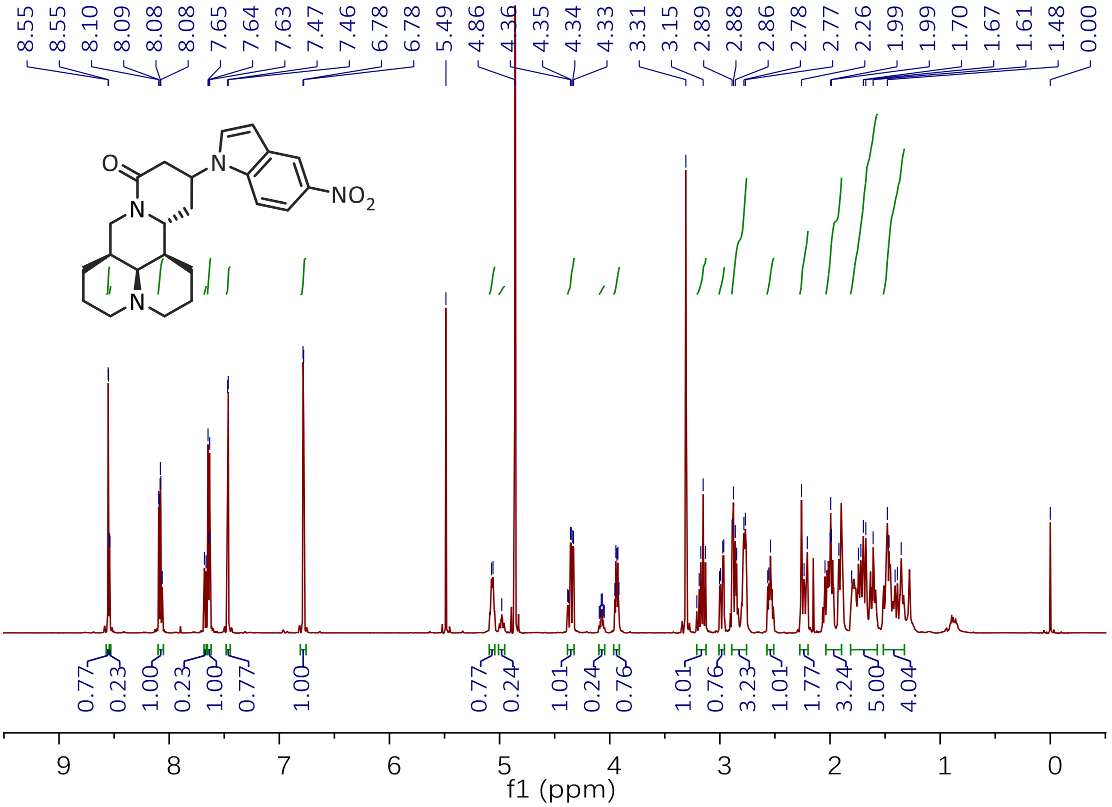

S13-2 ¹³C NMR spectrum of 3i (MeOD-*d*₄, 151 MHz)

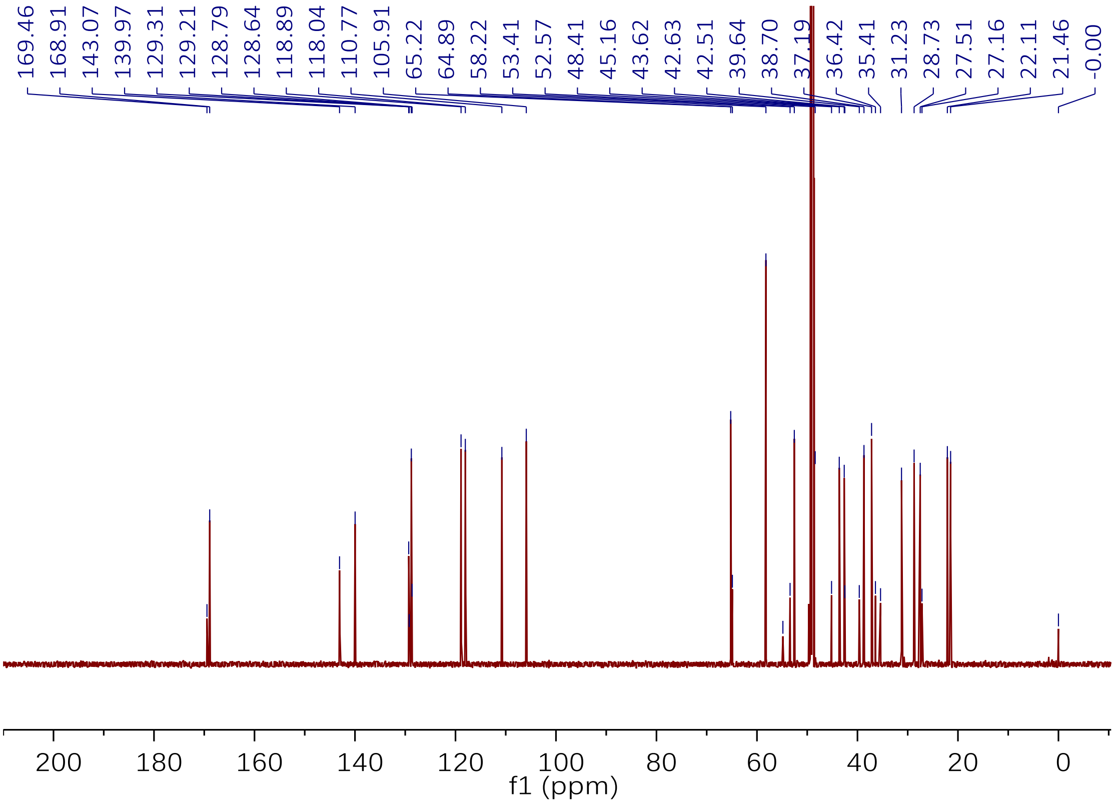

S14-1 ^{1}H NMR spectrum of 3j (MeOD-*d*4, 600 MHz)

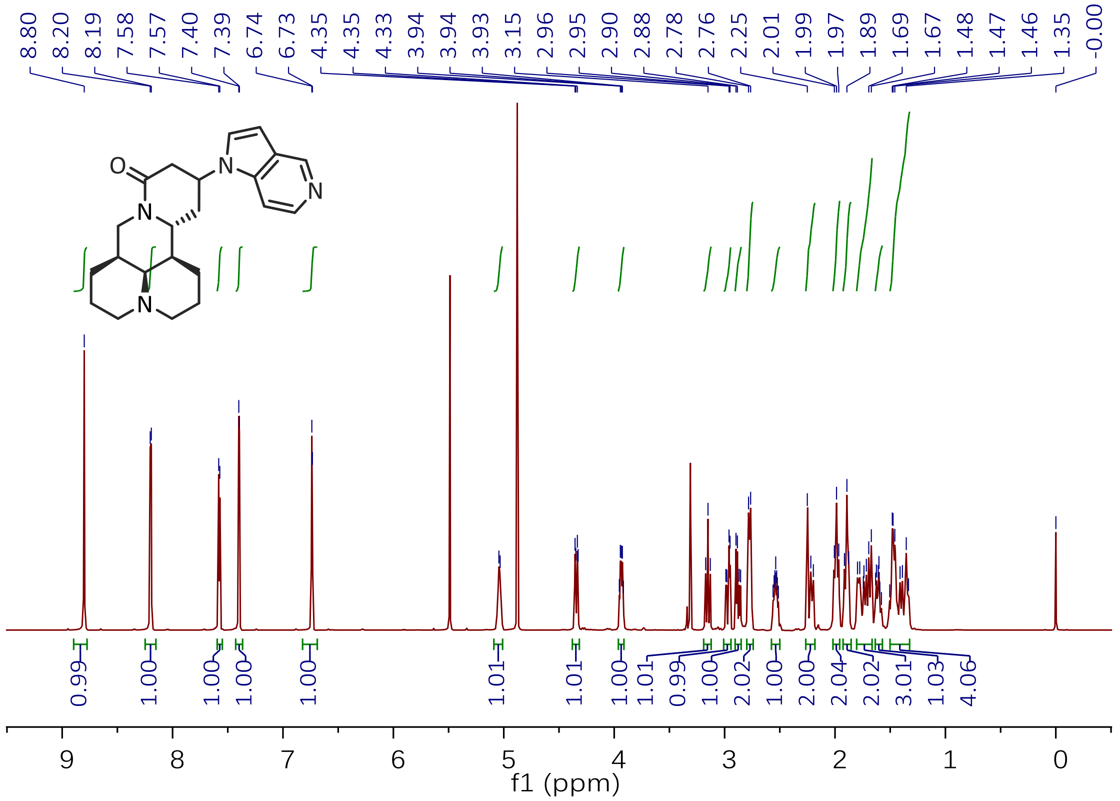

S14-2 ^{13}C NMR spectrum of 3j (MeOD-*d*4, 151 MHz)

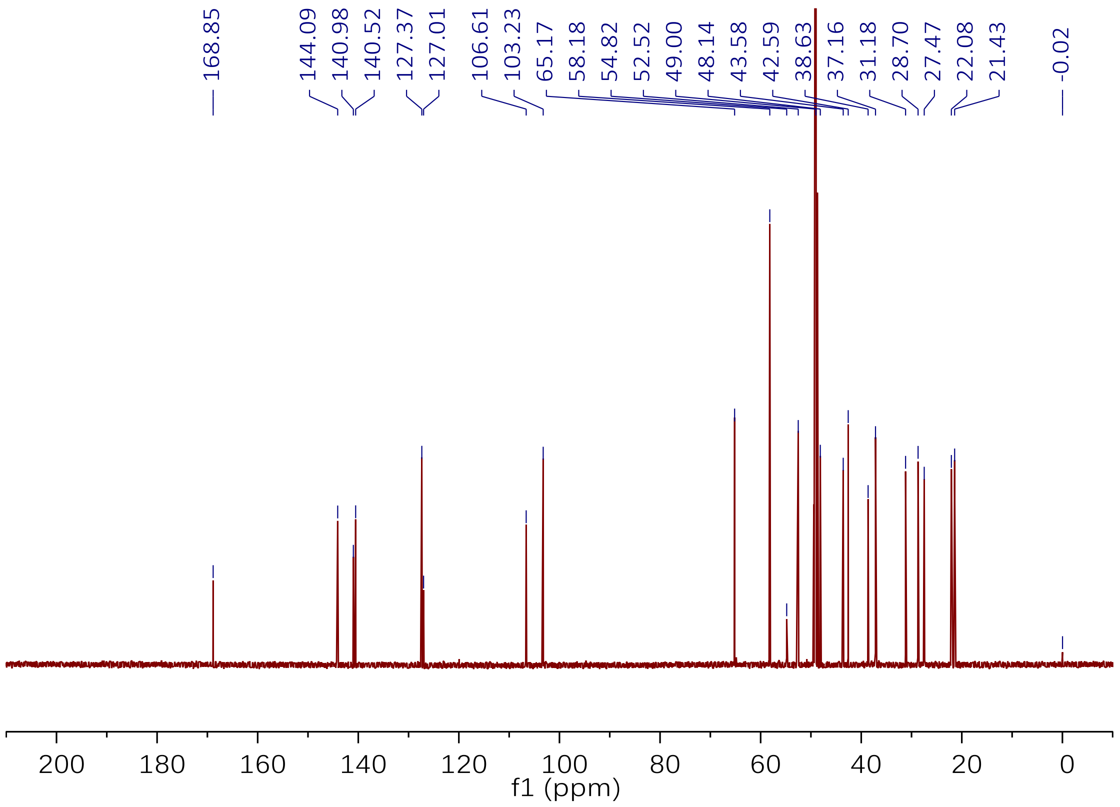

S14-3 NOESY spectrum of 3j ($CDCl_3$, 600 MHz)

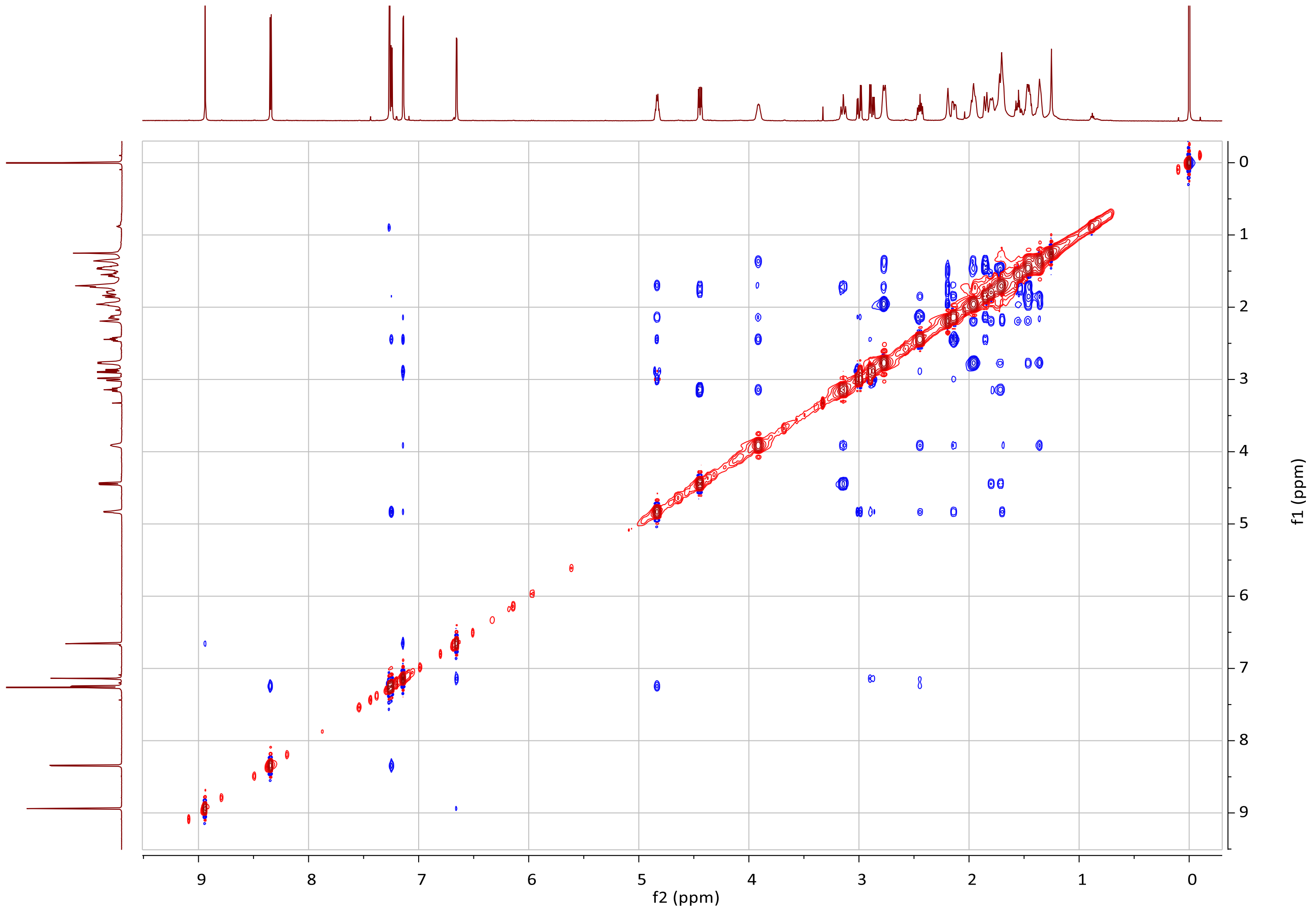

S15-1 ^{1}H NMR spectrum of 3k (MeOD-*d*$_4$, 600 MHz)

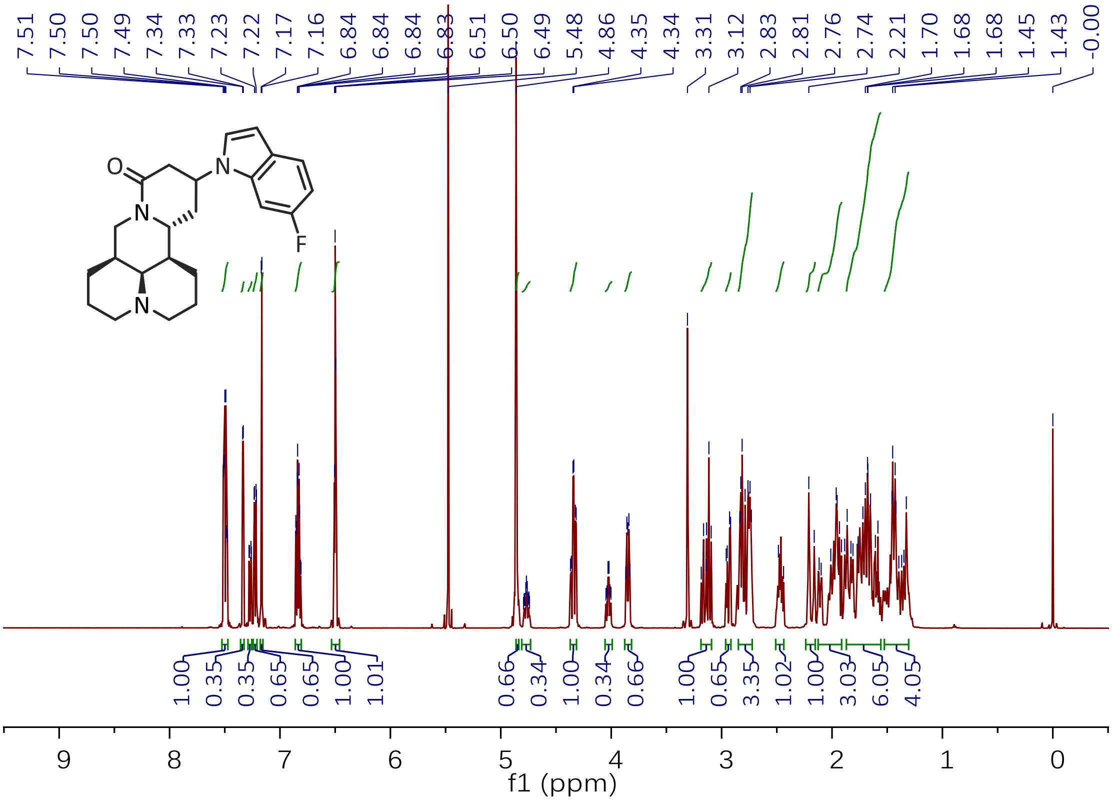

S15-2 ^{13}C NMR spectrum of 3k (MeOD-*d*$_4$, 151 MHz)

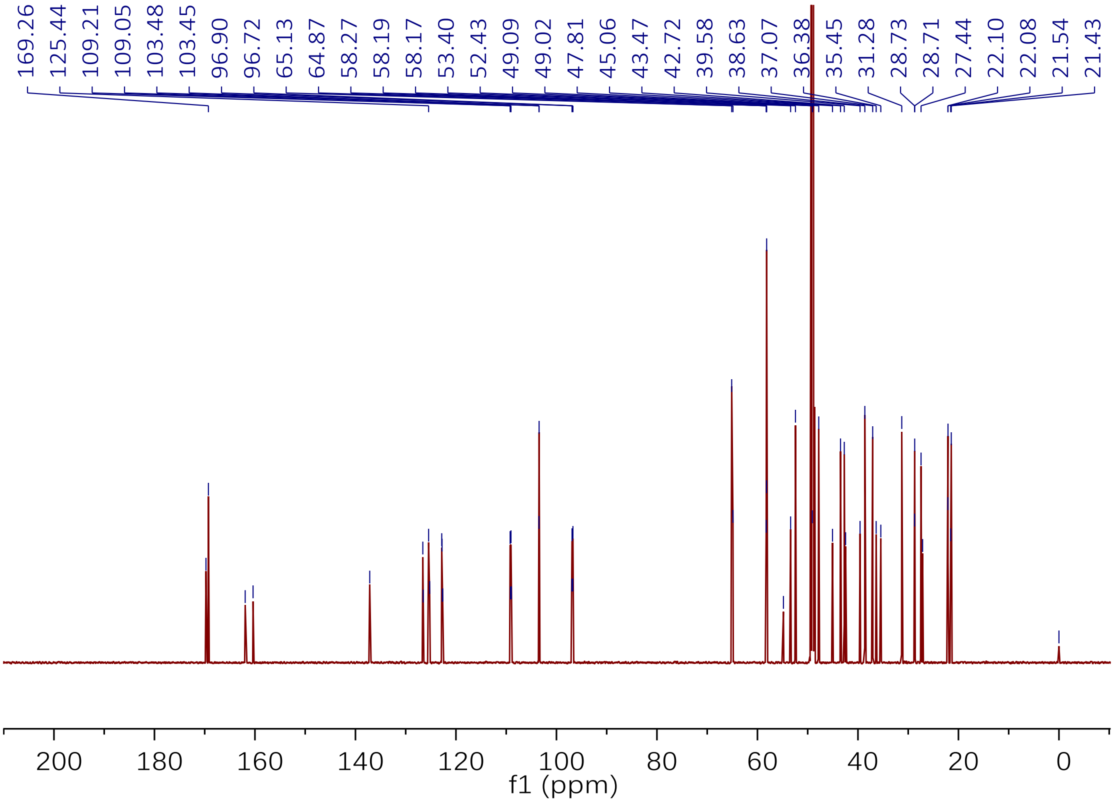

S16-1 ^{1}H NMR spectrum of 3l (MeOD-d_4, 600 MHz)

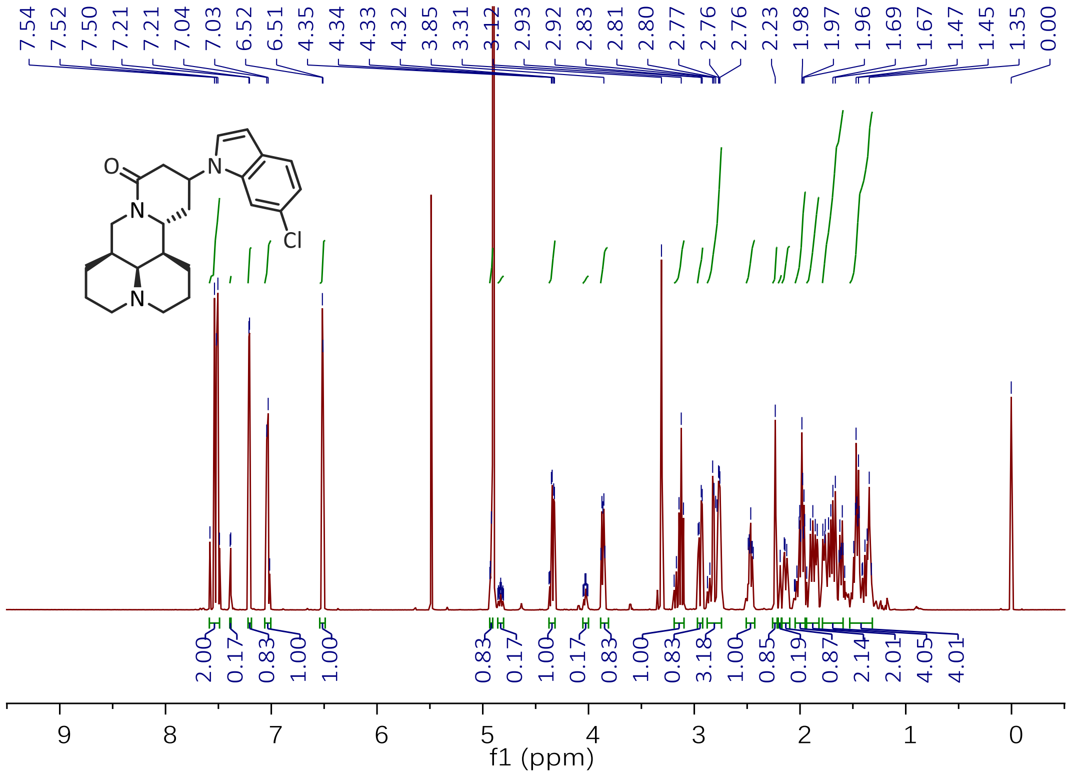

S16-2 ^{13}C NMR spectrum of 3l (MeOD-d_4, 151 MHz)

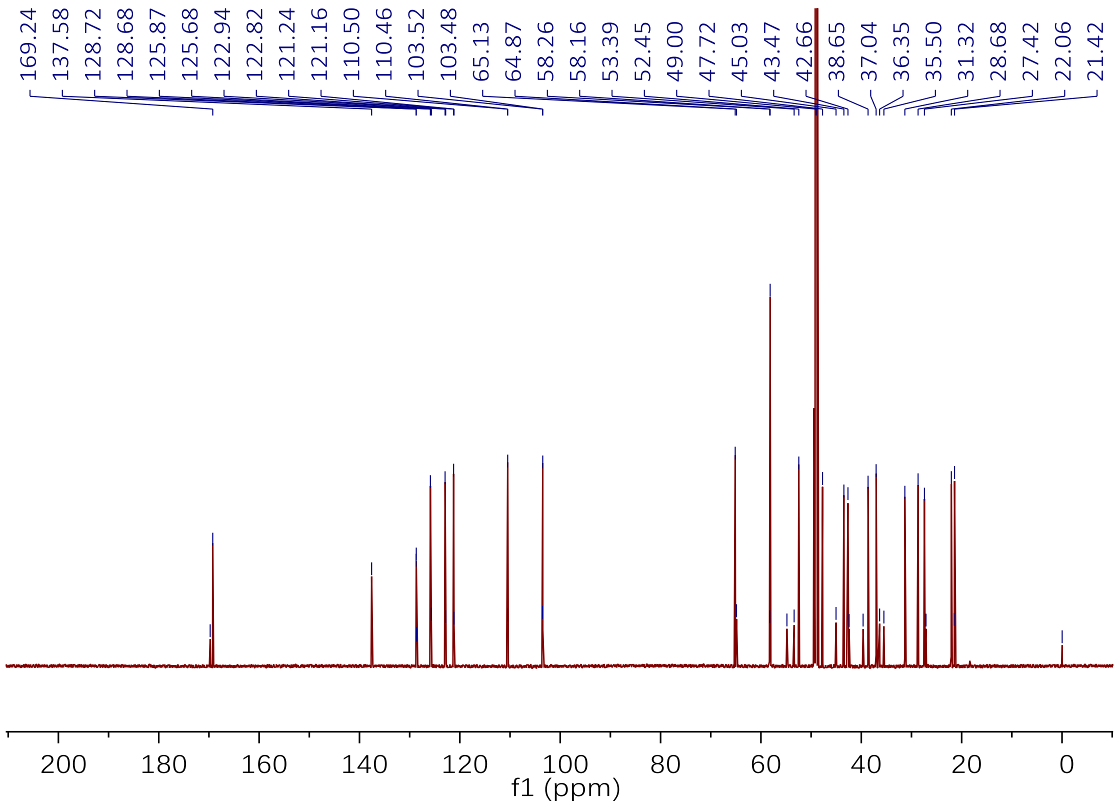

S17-1 ^{1}H NMR spectrum of 3m (MeOD-d_4, 600 MHz)

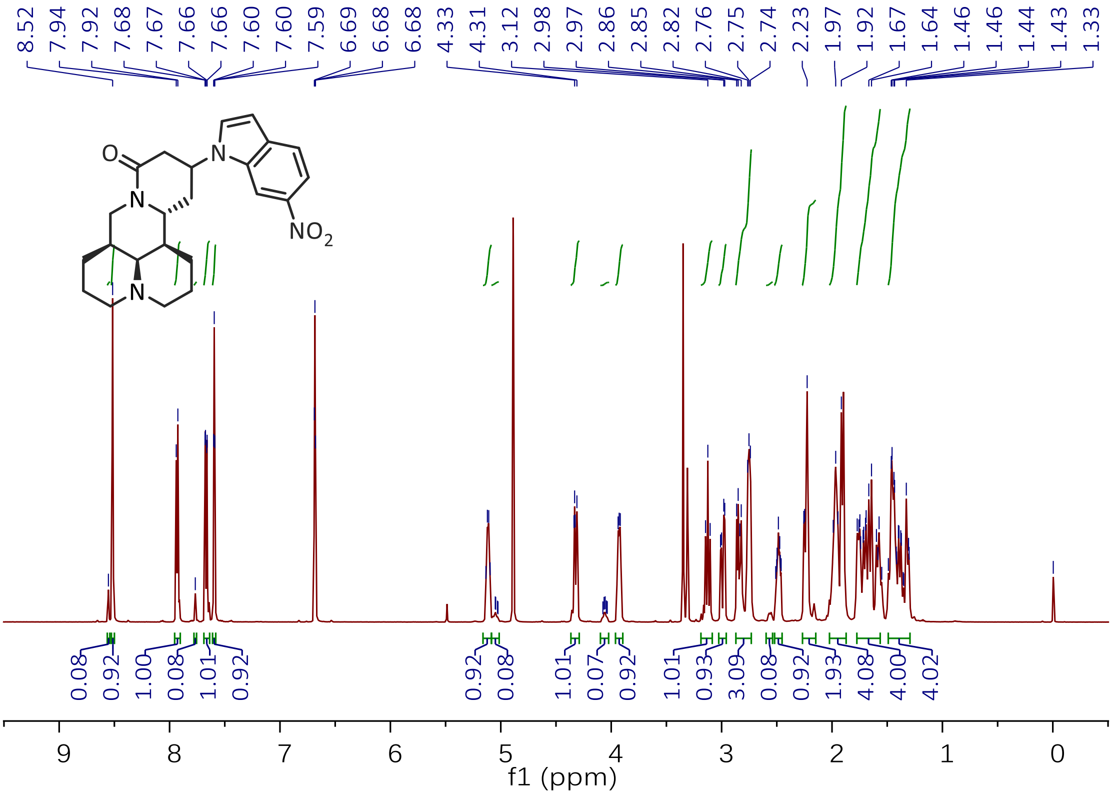

S17-2 ^{13}C NMR spectrum of 3m (MeOD-d_4, 151 MHz)

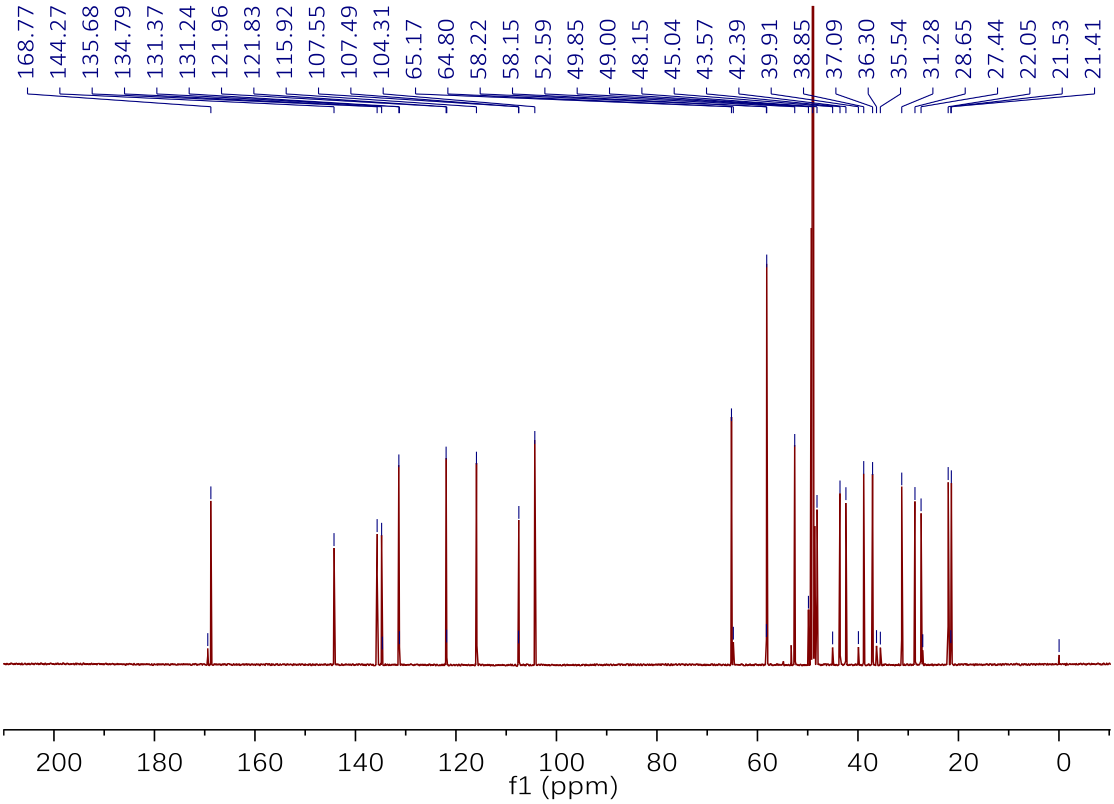

S18-1 ^{1}H NMR spectrum of 3n (MeOD-d_4, 600 MHz)

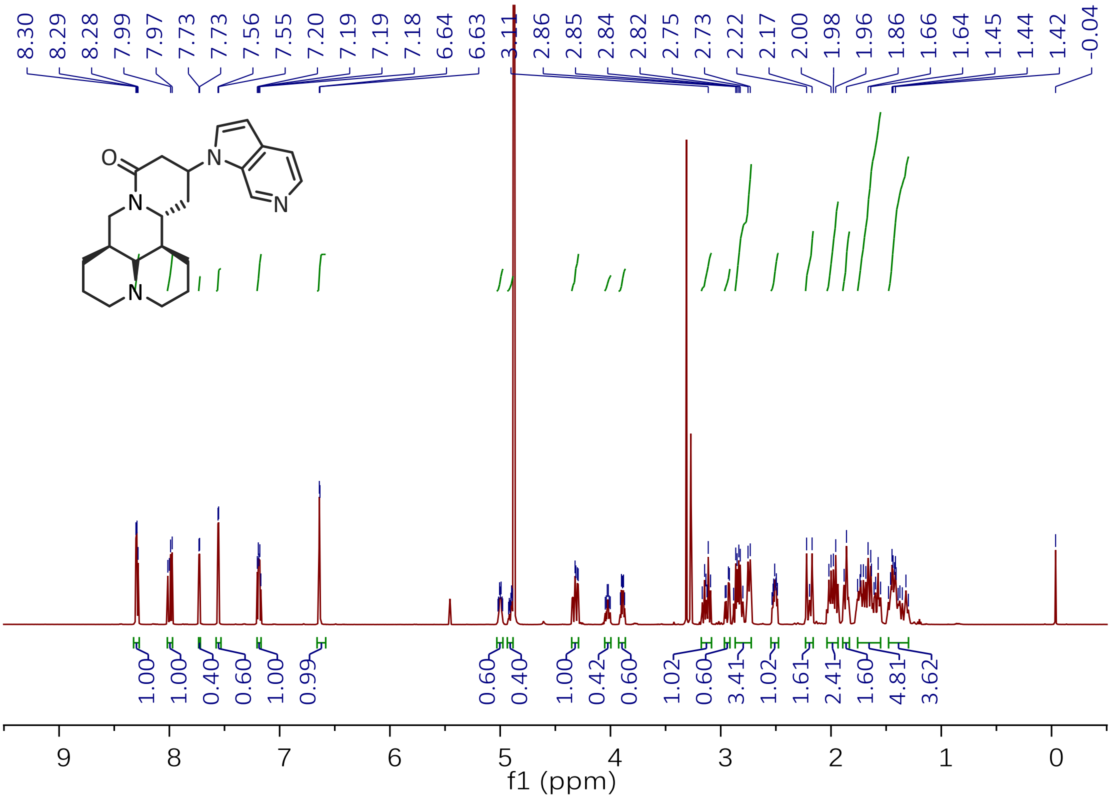

S18-2 ^{13}C NMR spectrum of 3n (MeOD-d_4, 151 MHz)

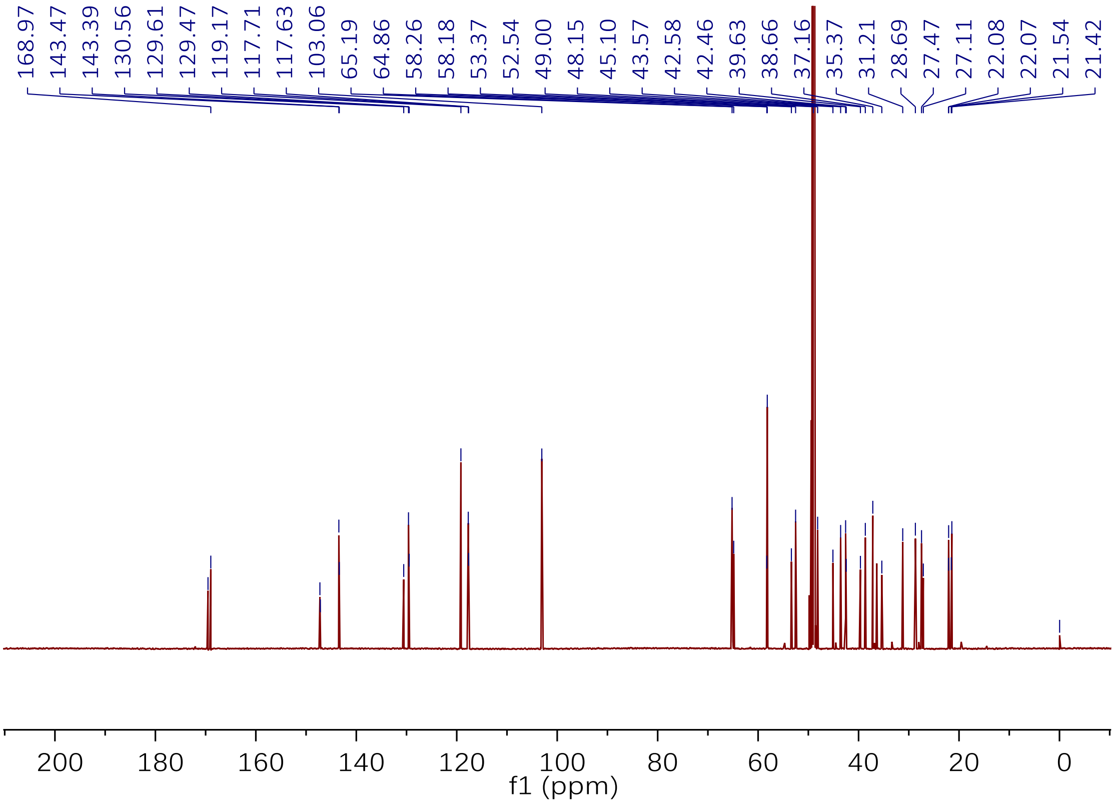

Supplement: Supplementary file 1 [file molecules-24-01108-s001.pdf]
